# Alkaloids of the Genus *Datura*: Review of a Rich Resource for Natural Product Discovery

**DOI:** 10.3390/molecules26092629

**Published:** 2021-04-30

**Authors:** Maris A. Cinelli, A. Daniel Jones

**Affiliations:** Department of Biochemistry and Molecular Biology, Michigan State University, East Lansing, MI 48824, USA

**Keywords:** alkaloid, Solanaceae, tropane, indole, pyrrolidine, *Datura*

## Abstract

The genus *Datura* (Solanaceae) contains nine species of medicinal plants that have held both curative utility and cultural significance throughout history. This genus’ particular bioactivity results from the enormous diversity of alkaloids it contains, making it a valuable study organism for many disciplines. Although *Datura* contains mostly tropane alkaloids (such as hyoscyamine and scopolamine), indole, *beta*-carboline, and pyrrolidine alkaloids have also been identified. The tools available to explore specialized metabolism in plants have undergone remarkable advances over the past couple of decades and provide renewed opportunities for discoveries of new compounds and the genetic basis for their biosynthesis. This review provides a comprehensive overview of studies on the alkaloids of *Datura* that focuses on three questions: How do we find and identify alkaloids? Where do alkaloids come from? What factors affect their presence and abundance? We also address pitfalls and relevant questions applicable to natural products and metabolomics researchers. With both careful perspectives and new advances in instrumentation, the pace of alkaloid discovery—from not just *Datura*—has the potential to accelerate dramatically in the near future.

## 1. Introduction

Perhaps no plants on Earth have been more famous—and infamous—throughout history than those in the genus *Datura.* Naturalized throughout the temperate regions of the world, *Datura* plants, like their other relatives in the family *Solanaceae*, are a rich source of bioactive phytochemicals. Although phenolics, steroids, acylsugars, amides, and other compounds from these plants have been isolated and characterized, it is the *alkaloids* found in *Datura* that have cemented these plants’ role in the remedies, religions, history, and lore of different cultures in all corners of the world.

As some of the tropanes, pyrrolidines, indoles, and other alkaloids present in the plant yield both medicinal use and insidious toxicity, *Datura* has also been the subject of fairly intensive study. For over a century, a complex, multidisciplinary effort with contributions from drug discovery and pharmacology, plant breeding and bioengineering, evolutionary biology, analytical chemistry, and ethnobotany has sought to elucidate the peculiar and powerful properties of these plants and the substances within that cause them. This comprehensive review covers the alkaloids of *Datura*, the tools used to detect and identify them, the factors affecting their content and composition, and challenges facing the plant natural products field as a whole. This information could aid anyone interested in discovering novel alkaloids or studying plant breeding or engineering systems for alkaloid production.

## 2. History and Taxonomy

### 2.1. Medicine and Culture

The historical significance of *Datura* is both medicinal and cultural. *Datura* plant parts, extracts, and preparations have been used as medicines by humans for millennia. Although comprehensive analysis of the medicinal uses of *Datura* is beyond the scope of this article, the reviews of Maheshwari [1], Benitez [2], and Batool [3] offer additional insights on this topic. *Datura* is described in ancient Chinese, traditional African, and Ayurvedic medicine. These plants were also used medicinally by indigenous Americans, and their properties were also described in Medieval European texts. *Datura* was a remedy for many conditions including pain, bruises, wound infections, swellings and boils, rheumatism, and toothaches, and *Datura* cigarettes were smoked to alleviate asthma and breathing problems [1,2]. Extracts and compounds (both alkaloids and other components) derived from *Datura* have also been investigated for, among others, anticancer, antibacterial, antiviral, and antifungal activities [1,3].

**Figure 1:**
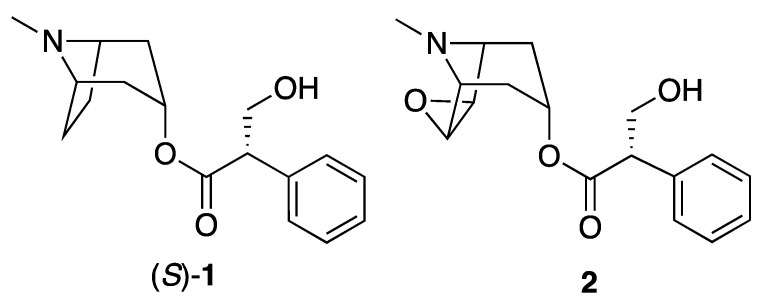
Hyoscyamine [(*S*)-1, the *S*-enantiomer of atropine, (**1**) and scopolamine (**2**), the most well-known alkaloids of the genus *Datura*.

The genus’ most famous alkaloidal constituents are the tropane alkaloids atropine (racemic **1**, or hyoscyamine, (*S*)-**1**), see below for clarification) and scopolamine (**2**, or hyoscine), which are shown in Figure 1. Tropane alkaloids (bicyclic [3.2.1] alkaloids) are known across many plant genera beyond *Datura*, and it is worth noting that the primary commercial sources of atropine and scopolamine are usually the genus *Duboisia* (and sometimes *Brugmansia*), and not *Datura* [4]. Alkaloid **2** is a muscarinic antagonist used to treat nausea, vomiting, and motion sickness, whereas atropine (**1**) is a similar anticholinergic agent, and is used in the treatment of certain poisonings and heart conditions, and to dilate the pupils in ophthalmology. Both of these drugs are listed on the World Health Organization’s Model List of Essential Medicines [5], and synthetic or semisynthetic derivatives of these drugs are also used.

As *Datura* contains high levels of these potent alkaloids (and many others whose bioactivity is ill-defined), all parts of the plants are considered toxic, and can easily poison both humans and livestock animals if accidentally or deliberately ingested [6,7]. *Datura* poisoning in humans causes dilated pupils, increased heart rate, blood pressure, and body temperature, dry mucous membranes, confusion, depression, and incoherence [8,9]. Severe symptoms can cause heart arrhythmias, coma [8], respiratory depression, and death. In one 26-year period in the United States, *Datura* species were responsible for 20% of fatal plant poisonings in humans, despite their consumption being uncommon [7]. *Datura* poisoning also causes characteristic delirium and hallucinations. Aggression, memory and recognition loss, picking at imaginary objects, delusions of being attacked by animals, public indecency, and other erosions of social inhibition have been reported [10,11,12,13,14]. Many names for *Datura* in different languages, such as *devil’s trumpet*, *malpitte* (Afrikaans, “crazy seeds”, and *torna-loca* (Spanish, “maddening plant”) reflect this particular property, and *Datura* has sometimes been used recreationally to induce these hallucinations. Due to their toxicity and ability to render subjects delirious and unaware, *Datura* species have also been used as a deliberate poison, especially to facilitate robberies or sexual assault [2,4,9,15].

*Datura* also has religious and cultural significance. As this is often the case in the same cultures where it is used medicinally, remedy, magic, tradition, and religion are sometimes difficult to separate. Indigenous American peoples brewed *Datura* into tea or chewed parts of the plant, utilizing its hallucinogenic and euphorigenic properties in initiation, divination, or luck-bringing ceremonies, or to bring skill or strength prior to hunting [16,17]. In archeological sites in the Southwestern United States and Central America, ceramic forms resembling the spiky fruit of *D. inoxia* (or a similar species) were found [17], and in California, chewed masses of *D. wrightii* were found alongside cave paintings believed to represent the flower of this plant [16]. Plants matching the description of *D. metel* appear in Hindu iconography depicting the god Shiva [18], while in Haiti, *D. stramonium* is associated with catatonia and zombies related to vodou [19]. In Europe, where *Datura* was believed to have been introduced by Roma immigrants, the plants, along with other toxic nightshades, became tied to witchcraft, the devil, and other dark or occult notions [12,15].

### 2.2. Description and Taxonomy

**Figure 2:**
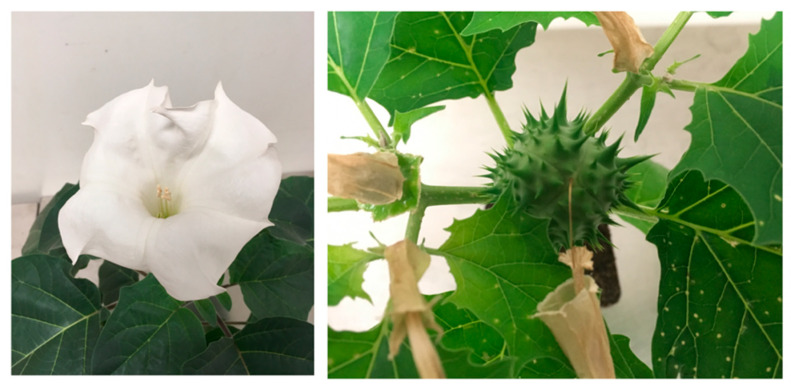
Flower of *D. metel* (**left**) and fruit of *D. stramonium* var. *stramonium* (**right**).

*Datura* species are herbaceous annuals or in some places, perennials. The plants are characterized by trumpet-shaped flowers, often malodorous foliage, and spiny fruits (excluding the smooth-fruited *D. ceratocaula*). The representative morphologies of *D. stramonium* and *D. metel* are shown in Figure 2. *Datura* is classified as a “nightshade” in the family Solanaceae, sub-family *Solanoideae*, and tribe Datureae. Datureae is comprised of four accepted sections, three of which belong to the genus *Datura*—*Dutra*, *Stramonium*, and *Ceratocaulis* [20]. The genus was originally known as Stramonium, but Linnaeus, in 1737, renamed it *Datura*, a likely Latinization of the Sanskrit *dhattura* [21], a word used to refer to the plant (probably *D. metel*) in classical texts.

There are generally considered to be nine species of Datura, although anywhere from 9–15 are reported in the literature, which can lead to confusion. *Datura stramonium* (jimsonweed, section *Stramonium*) is naturalized throughout the world. This species has four varieties (vars. *stramonium*, *tatula*, *godronii*, and *inermis*) that differ in their flower and fruit color and morphology [22]. Clustered in the same section (*Stramonium*) as Jimsonweed is *Datura ferox* (fierce thornapple). *Datura quercifolia* (from the Latin “oak-leaved”) is a shrub-like, American *Datura* species that is not extensively studied, much like *Datura discolor*. Closely related to *D. discolor* is *D. inoxia* (often spelled *innoxia*, Latin for “innocent” or “innocuous”), which is native to and found throughout the Americas. *Datura wrightii*, (“Wright’s *Datura*” or “Sacred *Datura*”), is often polymorphic, but it is distinguishable from *D. inoxia* [23]. Another plant related to *D. inoxia* and *D. wrightii* (and also in section Dutra) is *D. metel*, a cultivated ornamental with many varieties, including *alba*, *fastuosa*, *rubra*, *metel*, and *muricata* [24]. *Datura leichhardtii* (section *Dutra*) has two subspecies (*leichhardtii* and ssp. *pruinosa*) and is characterized by its small, yellowish-white flowers. *Datura ceratocaula* (from the Latin “horn-stalked”) is an aquatic, hollow-stemmed *Datura* species native to Mexico and South America and is the lone member of section *Ceratocaulis*.

*Datura* taxonomy is notoriously confusing. Several other described species were later reclassified. For example, the name *D. meteloides* is ambiguous; it is usually classified as *D. wrightii*, but occasionally as *D. inoxia* [6,25,26]. *D. lanosa* was reclassified as either *D. wrightii* or *D. inoxia*, whereas *D. velutinosa* and *D. guyaquilensis* are generally accepted to be *D. inoxia* [6,25]. *D. kymatocarpa* and *D. reburra* are usually described as conspecific with *D. discolor*, although phenetic comparisons suggest that they are actually all disparate species [27]. Additionally, different varieties of a single species are sometimes classified as separate species (e.g., *D. fastuosa* for *D. metel* var. *fastuosa* or *D. tatula* for *D. stramonium* var. *tatula*). Additionally, members of the related arboreous South American genus *Brugmansia* (the fourth section of the tribe *Datureae*) were previously classified in the genus *Datura*. but these two genera have been accepted as morphologically distinct since the 1970s [28].

## 3. Alkaloid Isolation and Purification, and Analytical Techniques for Detection, Quantification, and Identification

Before carrying out further discussion of the structures or origin of *Datura* alkaloids, it is helpful to review both the methods used to isolate and/or purify them from plants, and the techniques and instrumentation used to detect or quantify alkaloids and gain structural insights about the molecules themselves. The natural products field is beholden to analytical chemistry, and the pace of alkaloid discovery has accelerated dramatically since the 1950s with the development of modern instrumentation and advent of new methodologies in chromatography and spectroscopy. The following section applies only to *Datura* and its alkaloids; excellent reviews for more general alkaloid analyses, or analysis of those from other genera, have been written by Christen [29,30] and Petruczynik [31].

### 3.1. Extraction and Purification of Alkaloids

Extractions of alkaloids from *Datura* plant tissues have been performed on fresh, flash-frozen, or dried *Datura* tissue. Tissue is often macerated or powdered (sometimes mechanically, such as the ball-milling technique used by Moreno-Pedraza [32]), and then treated with an appropriate solvent to extract the alkaloids. Most solvents described in the literature are relatively non-reactive (i.e., will not degrade the components or significantly change the chemical composition of the sample). Examples include methanol [33] (though extractions with 100% methanol may lead to artifactual reactions, particularly with aldehydes [34]), methanol/water or ethanol/water mixtures [35,36], 5% concentrated ammonium hydroxide in ethanol [37], or ethyl acetate (following alkalization) [38]. Some reports describe the addition of acid to the solvent, as alkaloids are more soluble in water or polar solvents when protonated: Witte [39] used 2 M hydrochloric acid, while Doncheva and colleagues [40] used 3% sulfuric acid, although strong acidic and basic conditions present a risk that reactive groups may decompose during extraction.

Samples are sometimes heated or agitated to facilitate extraction. Temerdashev [41] compared different extraction methods for extracting *D. metel*, including those with heating and shaking, and found that a 1:1 mixture of 0.1 M hydrochloric acid/70% ethanol in water, heated to 60 °C in a water bath, yielded maximum recovery of alkaloids hyoscyamine and scopolamine. Djilani et al. [42] also compared extraction methods, and found that classical room-temperature solvent extraction, Soxhlet (reflux-temperature continuous solid-liquid) extraction, and solvents containing surfactants did not dramatically differ in their efficiency of extracting alkaloids from *D. stramonium*. This should not come as a great surprise, since the known *Datura* alkaloids range from polar to semi-polar compounds soluble in a wide range of solvents.

Following removal of insoluble materials by filtration or centrifugation, extracts are analyzed (such as in GC-MS or LC-MS metabolic profiling studies) without further processing, but additional measures can be taken to remove potentially interfering non-alkaloid substances. If an acidic extraction medium is used, it may be extracted with a nonpolar solvent (such as hexanes) to remove nonbasic lipids [43]. Since many *Datura* alkaloids are usually organic-soluble in uncharged free base forms, polar aqueous extracts can be basified (with, for example, sodium hydroxide) and extracted with a semipolar solvent such as chloroform [43], dichloromethane [44], or ethyl acetate [45]. More common is the application of the basic extract to solid-phase extraction columns, where the alkaloids are then eluted with dichloromethane or a similar solvent, as described by Berkov and colleagues (see below). In a recent report, Ciechomska [46] and colleagues developed a highly replicable, microwave-assisted extraction followed by solid-phase extraction to isolate atropine and scopolamine from *D. metel*, a technique which the authors report to consume less sample and less time than solid-liquid or Soxhlet extraction. It is worth noting that all published extraction methods should be approached critically. While certain conditions may work well for targeted compounds (such as **1** and **2**), other alkaloids may degrade under acidic conditions, whereas esters may be hydrolyzed under basic conditions, particularly at elevated temperatures. More labile alkaloids could potentially be missed in analysis because of harsh extraction conditions.

More specific purifications of individual alkaloids have been carried out using various methods, which include classical (usually normal phase) column chromatography, preparative or semi-preparative HPLC, preparative TLC, recrystallization, and other specialized stepwise protocols. Liu and colleagues [47] purified four indole alkaloids (**3–6**, Figure 12) from *D. metel* using silica gel column chromatography (eluting with chloroform/methanol), followed by preparative C_18_ HPLC and Sephadex column chromatography, to yield the indole alkaloids in 96.4–97.8% purity, although the yield was very low—only milligram quantities of the alkaloids were isolated from 7 kg of *D. metel* seeds. Welegergs et al. [48] purified a trisubstituted tropane alkaloid (β-**7**) from *D. stramonium* seeds using a combination of silica gel chromatography and preparative TLC; Siddiqui and co-workers similarly used preparative TLC (chloroform/ether) to isolate the nonpolar benzoyltropane datumetine (**8**) from *D. metel*, which they then recrystallized as a high-purity hydrochloride salt [45]. In a unique example of column chromatography, Beresford and Woolley first used a column of Kieselguhr (diatomaceous earth) buffered with phosphate (pH 6.8) to fractionate *D. ceratocaula* aerial part extract into three “bases” when eluting with light petrol followed by ether; alkaloid **8** (SI Table S1) was isolated from one fraction [49]. A method for isolation of high purity 2 (>99%) from “Hindu Datura” (presumably *D. metel*) flowers was recently reported by Fu [50]. Following several liquid-liquid extraction, basification, and back-extraction steps, the crude alkaloid residue was applied to a bed of D151 resin (a macroporous, cation-exchange resin), and then desorbed using an acidic aqueous solution (pH 1). Basification, desalting, and treatment with hydrobromic acid afforded crystalline scopolamine hydrobromide.

### 3.2. Crude Detection, Thin-Layer Chromatographic (TLC), Colorimetric, Densitometric, and Optical Methods

Early detections of *Datura* alkaloids employed colorimetric or spectrophotometric means, some of which are still employed today. Fuller and Gibson used the Vitali-Morin reaction on extracts of *D. stramonum* var. *tatula* [51]. In this reaction, the crude alkaloid extract was treated with nitric acid and then acetone in the presence of potassium hydroxide—the reaction of acetone with nitrated aromatic groups in alkaloids results in a red color, which can be quantified spectrophotometrically at 540 nm. This procedure is limited to aromatic alkaloids and, therefore, under-quantifies total alkaloid content. Alkaloids have also been titrated with sulfuric acid and bromocresol green [52], or with sulfuric acid and then back-titrated with sodium hydroxide and methyl red [53]. The Mayer reagent (mercuric chloride and potassium iodide) forms a white precipitate upon reaction with alkaloids; it has even been used for preparative scale alkaloid isolation from *D. stramonium* [42]. The most common reagent still used for alkaloid detection is the Dragendorff reagent (potassium bismuth iodide). This has broad reactivity for *Datura* alkaloids, including tropanes, indoles, and pyrrolidines, but it does not detect calystegines [54].

Alkaloids in crude mixtures can be separated and detected by TLC. Most frequently, silica-backed glass TLC plates are used. Visualization of alkaloids may use short-wave UV light, iodine [45], iodoplatinate or Dragendorff reagent, or densitometry and digital processing [55,56]. Advanced TLC methods have been published: Sharma et al. [55] employed high-performance TLC with an automatic TLC sampler and densitometric measurements following Dragendorff staining. This technique allowed the authors to determine alkaloid “fingerprints” for morphotypes of *D. metel* and quantify atropine and scopolamine, which separated very well using this method. Malinowska et al. [56] found that ETLC, or planar electrochromatography, was effective at resolving the alkaloids of *D. inoxia* [57]. Although TLC can be performed very quickly, it often has poor sensitivity: stain colors can fade, excessive background staining often occurs, and many components are not UV-active (or stain-reactive), making exact quantitative measurements very difficult. Duez et al. [58] however, described how staining TLC plates with *p*-dimethylaminobenzaldehyde (following development with a mixture of 1,1,1-trichloroethane and diethylamine) and using scanning densitometry resulted in alkaloid separation and quantification power comparable to HPLC, although it is worth noting that this report was published before the advent of many modern HPLC instruments.

Although it is uncommon, infrared (IR) spectroscopy has been applied to *Datura* alkaloids. Alkaloids (*S*)-**1** and **2** have characteristic IR bands at 1732 and 1170 cm^−1^ (indicative of esters, with the former being the carbonyl C=O stretch), 635 and 696 cm^−1^ (out-of-plane monosubstituted aromatic bending), and 858 cm^−1^ (scopolamine epoxide). By examining these bands in the ATR-FTIR (attenuated total reflectance Fourier transform IR) spectra of leaf powder of several nightshade genera (*Atropa*, *Datura*, *Duboisia*, *Hyoscyamus*, and *Solanum*), Naumann and colleagues were able to group similar spectra and chemotaxonomically organize these genera, as well as develop calibration curves for quantifying **1** and **2** in nightshades [59]. Ultraviolet (UV)-visible spectroscopy, by contrast, is commonly employed, and almost always in tandem with HPLC, although the UV–VIS spectra of individual compounds are sometimes collected as part of characterization [47]. Monitoring is usually performed at a wavelength of 210 nm, as most UV-active functionalities, including carbonyl groups, absorb in this region [60]. Wavelengths of 254 or 259 nm are also used, although these are more specific for aromatic alkaloids [35,58]. Two drawbacks of monitoring via UV–VIS spectroscopy are (a) that some alkaloids do not have functional groups that absorb UV light in a useful wavelength range, so they cannot be detected by this method; and (b) other compounds containing UV-active functional groups (e.g., phenolics, unsaturated fatty acids, amino acids, steroids, and terpenes) can interfere, especially if chromatographic resolution is poor.

As most alkaloids are chiral compounds, polarimetry (the rotation of plane-polarized light) is sometimes used in elucidation of absolute configuration as part of characterization. One elegant example is found in the work of Beresford and Woolley [49] who isolated alkaloid **9** (above and below) from *D. ceratocaula*. This alkaloid contains two chiral moieties (the tropane and the methylbutyric acid), which were first separated by hydrolysis of the ester bond. The dihydroxytropane was then esterified using tigloyl chloride, and polarimetry confirmed an identical specific rotation to that reported in the literature for (–)-3α,6β-ditigloyloxytropane, confirming the absolute stereochemistry of the tropane. Similarly, the acyl portion was confirmed as (+)-2-methylbutyric acid by polarimetry and comparison to the literature.

### 3.3. Gas Chromatography-Mass Spectrometry (GC-MS)

Mass spectrometry is extremely useful and powerful for alkaloid profiling, as many alkaloids, especially tropanes and pyrrolidines, lack useful UV or fluorescent chromophores but form characteristic fragment ions whose masses are diagnostic of their substitution patterns. Putative structures can be assigned from these fragment ions and with the aid of standards [on the basis of both their fragmentation and retention time (LC) or retention index (GC)]. GC-MS has been the most widely employed technique for studying *Datura* alkaloids. It has been used for assessing the impact of experimental variables on alkaloid content, examining the metabolites of transformed root cultures, discovering novel compounds, studying alkaloid biosynthesis or metabolism, metabolite profiling, and for chemotaxonomy [22,40,61,62,63,64].

Usually plant extracts or compounds are analyzed as-is, although pre-derivatization has been performed [38,41,65,66] by treatment with *N,O*-bis(trimethylsilyl)trifluoroacetamide (BSTFA) or *N*-methyl-*N*-trimethylsilyltrifluoroacetamide (MSTFA). These reagents convert free alcohols to less polar trimethylsilyl (TMS) ether derivatives, including those in alkaloids like (*S*)-**1** and **2**. Silylation improves thermal stability, improves chromatography, and reduces analyte polarity.

Columns used for GC alkaloid analysis typically contain nonpolar polysiloxane stationary phases such as SPB1 [37], SE-54 [67], HP-5, HP-1, HP-19091S-433, OV-1, or RTX-5 columns [22,38,41,65,66]. Mass spectrometry is almost always performed in GC/MS analyses using 70 eV Electron Ionization (EI) and forms true molecular ions (M^+^) from loss of an electron, though these often fragment extensively. An advantage of GC-MS is that fragment ion masses of alkaloids are often diagnostic for structural features. Some characteristic fragment ions for tropane, nortropane, epoxytropane, and pyrrolidine alkaloids from *Datura* are given in Table 1 [68,69]. For example, mass spectra of compounds containing a *N*-methylpyrrolidine ring contain a prominent fragment (often the base peak) at *m/z* 84, whereas EI mass spectra of tropane alkaloids often contain fragment ions at *m/z* 124, 113, 112, 96, 95, 94, 83, and 82 that reflect decorations on the tropane ring system. Positions of substitutions on tropane rings and the identities of side chains can often be deduced by analyzing both the abundances of certain fragment ions as well neutral mass losses from the M^+^ ion (Table 1). For example, disubstituted tropanes containing a free hydroxyl group at position 3 frequently exhibit a base peak at *m/z* 113 [40,70] but other disubstitution patterns yield a base peak in the spectrum at *m/z* 94. Compounds containing a tigloyl group show a characteristic neutral loss of 99 Daltons (Da), and those containing tropic acid dehydrate and lose formaldehyde via McLafferty rearrangement [37]. In addition, many alkaloid standards are commercially available, allowing for comparisons of retention times, retention indices, and reference EI mass spectra.

An excellent chemotaxonomic study performed by Doncheva and co-workers in 2006 investigated the alkaloid composition of twenty plant accessions belonging to all four sections of the tribe *Datureae*, and identified various alkaloids in roots and leaves [40]. Sixty-six tropane alkaloids were detected, with considerable variation among the minor alkaloids across all sections and species. *Brugmansia* could be easily distinguished from *Datura,* as the former contained high contents of 3,6-disubstituted tropanes and pseudotropine derivatives. Section *Brugmansia*’s alkaloid profile was similar to *D. metel* var. *fastuosa* (section *Dutra*) suggesting a relationship between those two sections, whereas section *Ceratocaulis’* profile was distinct (few alkaloids, especially epoxytropanes, in the roots) and on the basis of this analysis, should be considered separate from the rest of the sections.

Although commonly used, GC-MS has many limitations for metabolite discovery. First, nonvolatile alkaloids such as underivatized polar compounds, or those of high molecular weight, may not elute as intact molecules and may not be reliably detected [68]. Second, because GC-MS usually employs high temperatures in both the columns and injection ports, there is potential for thermal decomposition of some compounds. Glycosides, *N*-oxides, or other sensitive functionalities can be “burned off” in injection ports, and dehydrations, eliminations, and intramolecular reactions occur frequently for such compounds. Some compounds frequently identified in *Datura* samples (see below) may be formed during GC-MS, and whether they are purely artifactual or actually occur *in planta* [39,61,64] is the subject of ongoing dispute. Therefore, quantitative analyses using GC-MS may overrepresent certain alkaloids while underrepresenting others, and using another technique (such as LC-MS) for validation may be beneficial. Another caveat of mass spectrometry (including GC-MS) is that fragment ion masses provide virtually no information about absolute configuration (i.e., enantiomers) unless a chiral GC (or LC) column or chiral derivatizing reagent is used.

### 3.4. Liquid Chromatography-Mass Spectrometry (LC-MS) and LC-Tandem Mass Spectrometry (LC-MS/MS)

High-performance liquid chromatography-MS (HPLC-MS) and ultrahigh-performance liquid chromatography (UHPLC-MS), are increasingly used as alternatives to GC-MS. LC-MS has been used to quantify alkaloids in *Datura* nectar [74], track the effect of water volume [32], geography and natural selection [75], herbivory, and plant age [76] on alkaloid content and composition, for investigating how animals metabolize *Datura* components [77], for historical investigation [16], and for general metabolic profiling [36,78,79]. LC-MS methods employ a solid stationary phase (column) and a high-pressure (HPLC; <6000 psi, UHPLC; to ~15,000 psi) delivery of a liquid mobile phase, which the analytes are dissolved in as they move through the column. An advantage of LC-MS is that it can be performed at lower (ca. 35–50 °C) and ambient temperatures, and avoids thermal degradation. A downside of LC-MS is that LC separations are less efficient than capillary GC separations, though a wider range of experimental parameters are available including a variety of mobile phase options, and exploration of the range of conditions needed to resolve analytes can be time-consuming. As in GC, enantiomers cannot be identified or differentiated unless chiral stationary phases are used (vide infra).

Reverse-phase C_18_ (octadecyl-capped silica)-based columns are frequently used for analysis and separation of *Datura* alkaloids. A pentafluorophenyl phase column has also been used to analyze tropane alkaloids [10] following the recognition that this column type is more retentive toward positively-charged analytes [80,81], and can be used in both reversed-phase and hydrophilic interaction chromatography (HILIC) modes. UHPLC columns [36,77] have also been used for profiling of alkaloids; UHPLC columns have smaller particle sizes to provide more efficient separations, often with shorter analysis times. Common polar mobile phases for HPLC-MS and UHPLC-MS include water/methanol or water/acetonitrile, with formic acid (0.1–3%), acetic acid, or ammonium formate [82,83,84] added. These additives serve to keep basic alkaloids protonated, which reduces chromatographic streaking and tailing but also reduces retention on reversed phase separations.

Profiling of *Datura* alkaloids using LC separations coupled to mass spectrometry (LC-MS) has been performed on a variety of mass spectrometry platforms including triple-quadrupole [10], ion trap [65], hybrid triple-quad/linear ion trap [36], time-of-flight (ToF) [75], quadrupole time-of-flight (QToF) hybrid, or orbitrap instruments [32,36,77,85]. Ionization usually has involved electrospray ionization (ESI), although APCI (atmospheric pressure chemical ionization) has also been used [16,84].

By ESI, most alkaloids are highly ionizable and give [M+H]^+^, or sometimes [M+Na]^+^, [M+K]^+^, or [M+H+CH_3_CN]^+^ pseudomolecular ions [76,83] with minimal formation of fragment ions. Alkaloid discovery is aided when LC-MS uses instruments with high mass resolution that provide accurate mass measurements that can distinguish isobaric compounds (i.e., those with common nominal masses but different elemental formulas), whereas experiments that employ more energetic ion activation methods (such as MS^E^) yield abundant fragments [86].

Tandem mass spectrometry (MS/MS), where a given ion is first selected (for example, by the quadrupole filter of a QToF instrument, and then fragmented using collision-induced dissociation) is an incredibly powerful technique for gaining structural insights into alkaloids and aiding in annotation by associating fragment ions with specific precursor ions.

**Figure 3:**
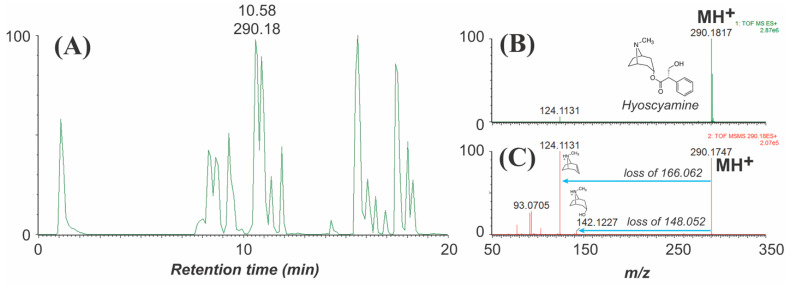
HPLC/ESI-MS base peak intensity chromatogram of a methanol-water extract of three-week-old *D. metel* root (left) using a C_18_ LC column on a Waters Xevo G2-XS QToF high-resolution mass spectrometer. The analysis was performed using data-dependent MS/MS in positive-ion mode. (**A**) Survey scan base peak intensity chromatogram. (**B**) The survey scan spectrum for hyoscyamine (retention time 10.58 min). (**C**) MS/MS product ion spectrum for *m/z* 290 for hyoscyamine shows characteristic monosubstituted tropane alkaloid fragment ions at *m/z* 142 and 124.

Much like the EI fragments obtained from GC-MS, ESI fragments (like those obtained by LC-MS) are also often diagnostic, but the ion fragmentation processes differ because EI-generated molecular ions are radicals, whereas ESI-generated ions are not. Some relevant positive-mode ESI fragment ion masses (from MS/MS) are also given in Table 1. For example, as observed in EI fragmentation, *N*-methylpyrrolidine-containing alkaloids frequently yield a *m/z* 84 fragment (*N*-methylpyrrolinium ion) or show neutral losses of 83 Da, whereas tigloyl esters are often eliminated as discrete units of 100 Da. Acyltropane substitution patterns are also easily differentiated by the fragments obtained when the acyl groups are lost as either neutral acids or ketenes/anhydro groups. An example of the fragment ions obtained from high-resolution ESI-MS/MS of hyoscyamine (detected in the chromatogram of *D. metel*) is shown in Figure 3.

Examination of the peak (*m/z* 290) in the chromatogram corresponding to hyoscyamine shows characteristic monosubstituted tropane fragments at *m/z* 124 and 142 [10,69]. The *m/z* 124 fragment results from neutral loss of tropic acid (166.063 Da theoretical mass), and loss of the anhydro-tropic acid fragment (148.052 Da) yields *m/z* 142; additional odd-mass fragments at *m/z* 93 and 91 result from loss of the nitrogen (as methylamine). The low mass defects (portion of mass that follows the decimal place) of the neutral losses derived from tropic acid suggest that these fragments are relatively hydrogen poor (e.g., aromatic). Although less predictable, fragments obtained from acylium ions (arising from elimination and dehydration of acyl groups) are sometimes visible and can provide information, along with the mass defects, about the nature of the acids esterified to the tropane core.

LC-MS has been used in remarkably diverse ways to characterize *Datura* metabolites. In one 2020 study, Hui and colleagues [36] used UHPLC-QToF MS/MS (positive-ion mode) to profile roots, leaves, stems, flowers, seeds and peels (possibly pericarp) of *D. metel*, and identified 65 alkaloids, which they classified as indole, amide, or tropane. This group also developed a UHPLC-Q-TRAP MS/MS technique to quantify 22 selected *Datura* metabolites (mostly hydroxycinnamic acid amides, although some *beta*-carbolines/indoles and tropanes were included). This method, employing a five-minute LC run, utilized multiple-reaction-monitoring (MRM) data acquisition. It was observed that seeds contained the highest amounts of the selected alkaloids; a dependence on geography was also observed.

An elegant example of HPLC-MS with a chiral column was described in a recent manuscript by Marín-Sáez [87]. In this method, a CHIRALPAK-AY3 column (silica support with amylose tris-5-chloro-2-methylphenylcarbamate, eluting with ethanol/0.1% diethanolamine), was used to separate the enantiomers of atropine. ESI-MS/MS was used to monitor the alkaloids, using the MRM transition *m/z* 290>124 characteristic of atropine/hyoscyamine. Excellent resolution of the two enantiomers was observed, and it was determined that *D. stramonium* seeds grown in Spain contained (–)-hyoscyamine (*S*)-**1**: (+)-hyoscyamine in a 90:1 ratio.

### 3.5. Other Mass Spectrometry Methods

In addition to GC-MS and LC-MS, newer methods have been developed with the goal of eliminating processing, extraction and chromatographic steps entirely. One such method is called direct analysis in real-time mass spectrometry (or DART-MS), where a whole tissue sample is inserted between the ion source and the mass spectrometer inlet. Molecules in the tissue are then protonated by water clusters (arising from carrier helium and atmospheric water), and, as in ESI, molecules more basic than water readily ionize. This method allows for rapid development of “heat maps” or “fingerprints” that show the spatial distribution of metabolites in whole tissues, which can rapidly identify plant species. Lesiak and Musah applied this method to *D. stramonium*, *D. inoxia*, and *D. ferox* seeds, which were sliced transversely and analyzed using soft-ionization positive-mode DART-MS [88]. Spectra of *Datura* seeds show characteristic tropane fragment ions (e.g., *m/z* 174, 158, 142, 124), and the different seed ions clustered on the basis of species by principal component analysis.

In another study by the same group [89], a method called Laser Ablation DART Imaging (LADI-MS) was developed. In this technique, a *Datura* seed was embedded in silicon putty on a laser imaging platform. A UV laser pulse then scanned over the sample, producing an analyte-rich ablation plume, which was carried through a tube using helium to the DART ion source-inlet space (as described above). As the composition of the ablation plume may differ spatially across the sample, the distribution of any given analyte can be effectively “imaged”. For example, in *D. leichhardtii* seeds, a different spatial distribution was observed for arginine (a biosynthetic precursor to tropanes), tropine (α-**10**), and hyoscyamine ((*S*)-**1**)/littorine (**11**, SI Table S1), which suggests the presence of sophisticated cellular transport machinery and/or specialized localization of alkaloid biosynthetic enzymes. Moreno-Pedraza elaborated on this technique by developing a UV laser desorption method that uses a low-temperature, 3D-printed plasma probe for sample ionization (known as laser-desorption-low temperature plasma mass spectrometry), which removes the need for sample matrices or organic solvents entirely. This technique was employed to image the distribution of tropane alkaloids and their fragment ions in cross sections of whole *D. stramonium* fruits and seeds [90].

Another method used for direct imaging is Desorption ESI (or DESI), where droplets of solvent are sprayed directly at an intact tissue sample or section, and the impact of the droplets produces gas-phase ions that are then analyzed. Cooks and colleagues [91] analyzed *D. stramonium* roots and seeds using this technique. With 1:1 methanol:water as the spray solvent, they were able to detect fifteen of the nineteen reported alkaloids in *D. stramonium* roots and confirm their identities by their fragment ion masses.

### 3.6. Nuclear Magnetic Resonance (NMR) Spectroscopy

NMR is frequently used for structure elucidation, and along with X-ray crystallography, is the most powerful tool for demonstrating molecular connectivity. Nonetheless, as the structures of many alkaloids (especially tropanes and pyrrolidines) are putative, proposed on the basis of mass spectral fragmentation (or comparison of retention time to standards), NMR is generally only used on isolated and purified alkaloids (see below).

**Figure 4:**
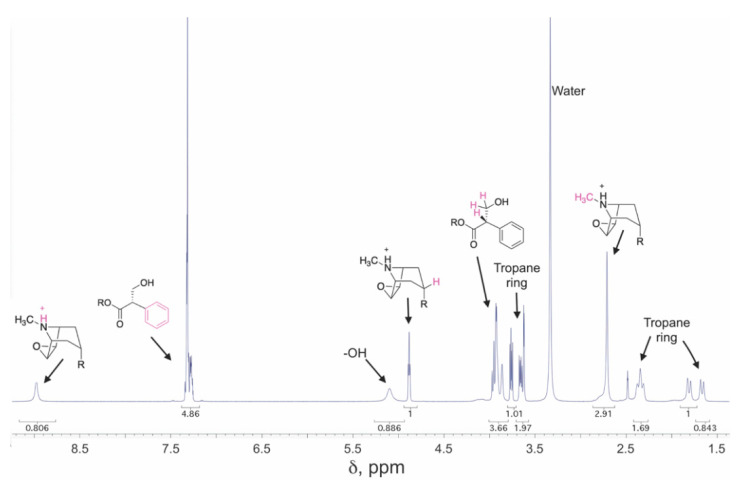
^1^H-NMR spectrum (500 MHz, in DMSO-*d*_6_) of scopolamine (**2**). Indicated peaks correspond to the structural features highlighted in magenta.

**Figure 5:**
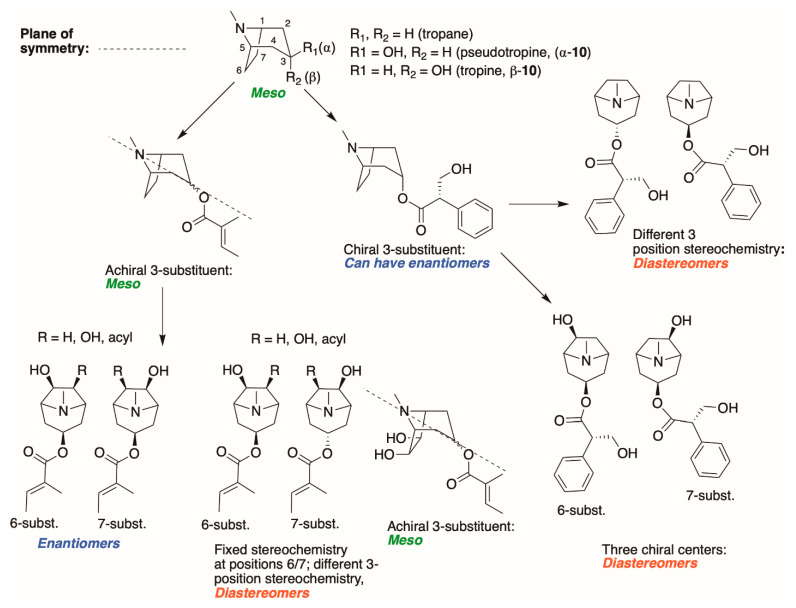
Chirality in tropane alkaloids and relationships between meso compounds, enantiomers, and diastereomers for substituted tropanes.

Typically, the first NMR experiment performed during structural elucidation is proton (^1^H-NMR) spectroscopy, which yields signals that reflect environments of the hydrogens (protons) in the molecule. ^1^H-NMR (or proton NMR) yields distinctive signals for the tropane nucleus [40], and analysis of the splitting patterns has been frequently used to determine the configuration at position 3 (e.g., α-**10**, or tropine-derived, or β-**10**, or pseudotropine, derived; see Figure 5). Additional information about groups esterified to the tropane core can be gained from examining the olefinic (5–7 ppm) and aromatic (6–8 ppm) regions. An example of a ^1^H-NMR spectrum of a tropane alkaloid (**2**) is shown in Figure 4. Carbon-13 (^13^C-NMR) spectra are also commonly obtained, although these experiments have lower sensitivity in part because NMR-active ^13^C nuclei are less abundant than protons. ^13^C spectra are also collected as part of DEPT (distortionless enhancement via polarization transfer) experiments. These experiments (DEPT-45, DEPT-90, and DEPT-135) distinguish ^13^C signals on the basis of the number of attached protons. In cases where the structure is more complex (as in many tropane alkaloids), two-dimensional NMR experiments that provide more explicit information about the connectivity of atoms, such as total correlated spectroscopy (TOCSY, H-H), correlation spectroscopy (COSY, H-H), heteronuclear single quantum coherence (HSQC), heteronuclear multiple-bond correlation (HMBC, H-C, or H-N), or various through-space experiments dependent on the nuclear Overhauser effect (such as NOESY or ROESY). Although the last experiments are not commonly included in the *Datura* alkaloid literature, they have been employed for structural determination of other tropane alkaloids [30].

The largest advantage of NMR spectroscopy is that, in addition to the myriad structural information it provides, it is generally nondestructive, and samples can be recovered after analysis. Nonetheless, it can be very time-consuming, especially when performing ^13^C-based or 2D-experiments on low field strength instruments, and its sensitivity is much lower than mass spectrometry. To perform structural determination of an alkaloid by NMR, generally 1–5 mg of a compound is required, which often makes isolation (and purification) of enough compound from a natural source a major barrier to NMR experiments. Additionally, as most alkaloids are chiral molecules, a downside of NMR, like mass spectrometry, is the inability of the method to establish absolute stereochemical configuration (e.g., enantiomeric identity or ratio)—that is usually established by chiral HPLC and comparison to a standard, circular dichroism or similar methods, X-ray crystallography, or chemical derivatization (although often not used for *Datura* alkaloids) [47,49,92]. However, in the presence of so-termed chiral shift reagents, enantiomers can be differentiated and quantified by NMR. Lanfranchi et al. [44] added one such reagent, Yb(hfc)_3_, to a solution of atropine trifluoroacetate. This reagent causes deshielding of certain carbons, and the splitting of certain ^13^C signals into discrete peaks corresponding to each enantiomer, from which enantiomeric ratio can then be determined. This method was optimized with different ratios of atropine enantiomers and then applied to crude *D. stramonium* seed extract, which revealed that (*S*)-(–)-hyoscyamine (*S*)-**1**) was the dominant enantiomer by a factor of 20:1 (in agreement with the chiral HPLC results, *vide supra*) [87].

NMR has also been used in studies of the metabolism and biosynthesis of alkaloids in *Datura* plants and root cultures derived from *Datura*. Robins et al. [63] fed *D. stramonium* root cultures with NMR-active ^13^C-labeled sodium acetate, hygrine (**12**, Figure 11), and several pyrrolidine esters, all of which could be hypothetical precursors of tropane alkaloids. By monitoring incorporation of the ^13^C label into tropane alkaloids by NMR, it was determined that hygrine and *N*-methyl-2-pyrrolidinyl acetate ester were not direct precursors of tropane alkaloids (hygrine was only found incorporated into cuscohygrine, **13**, Figure 11), but 1-methyl-2-pyrrolidinyl-3-oxobutanoate (see below) and sodium acetate likely were. ^15^N-NMR spectroscopy was also used to study the de-differentiation of *D. stramonium* root cultures fed with ^15^N-labeled nitrate or ammonium salts. When these cultures were treated with various hormones, the cells formed a suspension culture and lost most of their ability to synthesize tropane alkaloids. Instead, ^1^H-^15^N-HMBC spectra (see below) of extracts showed that these cells accumulated *gamma*-aminobutyric acid and *N*-acetylputrescine [62,93] suggesting that the nitrogen balance of these cells had shifted away from alkaloid biosynthesis.

The literature contains several examples of applying these experiments to the structural determination of alkaloids. Siddiqui et al. [45] isolated a *para*-methoxybenzoic acid tropane ester from *D. metel*, which was appropriately named datumetine (**8**). ^1^H-NMR revealed a triplet consistent with the methine of a 3α-tropyl ester and a characteristic downfield doublet-of-doublets suggestive of a *para*-substituted benzoate, which was confirmed by ^13^C-NMR. Additional information about the structure (such as the presence of methines indicative of a tropane bridgehead (2- and 5-positions) and the *para*-methoxyl group was revealed by DEPT. Similarly, Welegergs and colleagues isolated a trisubstituted tropane alkaloid from Ethiopian *D. stramonium* seeds (β-**7**), and they used a similar combination of ^1^H, ^13^C, and DEPT-135 NMR experiments to assign the structure of the isolated alkaloid as **7** (SI Table S3) [48]. Liu and co-workers utilized ^1^H-^1^H COSY and ^1^H-^13^C-HMBC, and ^1^H-^13^C-HSQC, in addition to ^1^H and ^13^C-NMR, to establish connectivity of the atoms in the daturametelindole alkaloids (**3–6**) isolated, obviously, from *D. metel*), several of which possess a unique spirocyclic oxindole core [47].

## 4. Alkaloids of the Genus *Datura*—Tropane Alkaloids

### 4.1. History—And Some Troublesome Aspects of Identifying and Reporting Tropane Alkaloids

Although the medicinal and psychotropic effects of *Datura* species have been known throughout history, the active alkaloid constituents were a mystery for centuries. Hyoscyamine [(*S*)-**1**)] and scopolamine (**2**) were not isolated from any plant until 1833 and 1881, respectively [6], and are named after *Hyoscyamus* and *Scopolia*—the genera from which they were first isolated. The principal alkaloid isolated from *D. stramonium* was originally called “daturine”. Daturine was later determined to be identical to two other alkaloids, “duboisine” (presumably isolated from the genus *Duboisia*) and hyoscyamine (which was established as being “chemically identical” to atropine, **1**). The observation that *Datura* actually contained (*S*)-**1** was made in 1880 [94], and appears to have been to be common knowledge by 1901 [95] but references to (*S*)-**1**′s possible presence in *Datura* existed as early as 1850! Although research on *Datura* alkaloids has been conducted for over 170 years, the majority of new alkaloids were identified from 1970 to the present.

There are several caveats that must be acknowledged when perusing the *Datura* alkaloid literature and examining the annotations and identifications of tropane alkaloids and the species that they are isolated from. First, several *Datura* species very closely resemble each other and may be polymorphic (i.e., *D. metel*, *D. wrightii*, and *D. inoxia*), which is complicated further by ambiguous or obsolete names in the literature (e.g., *D. meteloides*). Some of these similar species (such as *D. inoxia* and *D. wrightii*) also have significant overlap between their native or naturalized geographic ranges [23,25]. Without a definitive botanical or genetic identification of a wild plant accession, it may be difficult to tell exactly which species of *Datura* is being studied.

Second, not all *Datura* species have been studied equally with regards to alkaloids. Some species, like *D. stramonium*, have been profiled extensively using multiple analytical methods, while others, like *D. discolor* and *D. quercifolia*, are relatively uninvestigated. Although a specific alkaloid may be listed as absent from a certain species in the literature (or in SI Tables S1–S5), this may not mean that the alkaloid is truly absent; it may instead mean that it has not been discovered in that species to date, or the analytical methods used simply have not detected it.

Third, the tropane literature is rife with structural ambiguity. Tropane, tropine (α-**10**), and pseudotropine (β-**10**) are achiral *meso-*compounds that are bisected by an internal plane of symmetry (Figure 5) and many 3-monoacylated compounds are also *meso* compounds (unless they contain chiral acyl groups, like hyoscyamine (*S*)-**1**). Additional functionalization at position 6 or 7, however, can desymmetrize the molecule [92]. Therefore, 3,6- and 3,7-disubstituted tropanes (and some asymmetrically substituted 3,6,7-trisubtituted tropanes) form enantiomers. As substitutions on positions 6 and 7 (as far as those have been isolated) are always *exo* (β), asymmetrically substituted tropanes are only diastereomers if position 3 differs in its α/β configuration—see Figure 5. As mentioned above, enantiomers cannot be differentiated on the basis of achiral LC-MS, GC-MS, or standard NMR techniques used for structural elucidation. Since the majority of *Datura* alkaloids have been annotated based on GC-MS data, there is considerable uncertainty regarding absolute configurations. Stereochemistry often seems to be assigned arbitrarily, or by comparison to retention indices of standards whose structures themselves are not definitively assigned. In the following sections (and the tables and figures referenced herein), the structures of tropane alkaloids are described as reported by the original authors, although the stereochemical assignments may not have been definitively established.

For the purposes of brevity, classes of tropanes (organized into subgroups roughly following the conventions of Lounasmaa and Tamminen [96]) that contain many different compounds (monosubstituted, disubstituted, trisubstituted and epoxytropanes, and nortropanes) are organized into SI Tables S1–S5 (in the Supporting Information). It may be helpful for the reader to refer to these tables frequently while going through the following sections, as alkaloids are referenced in the text using numerical descriptors, some of which are only found in these tables. The structures of other alkaloids of note are depicted in Figures 7–13 (*below*).

### 4.2. Biosynthesis of the Tropane Core

**Figure 6:**
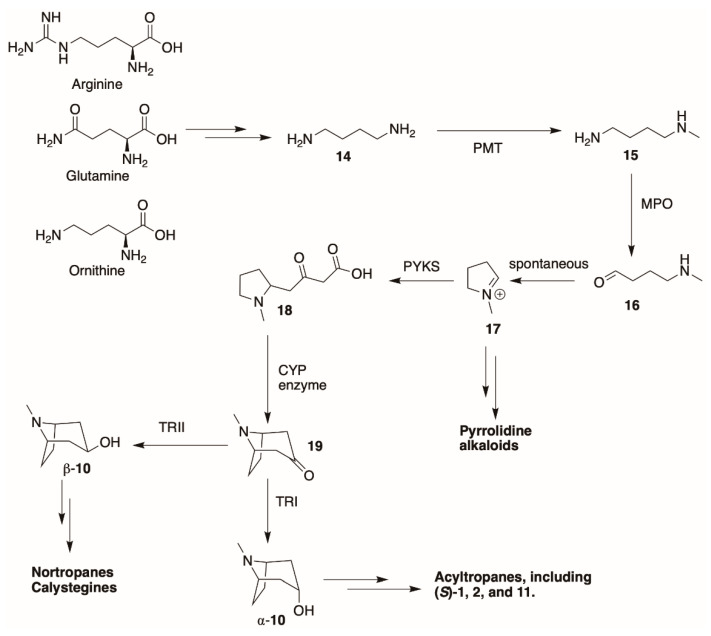
Biosynthesis of tropane and pyrrolidine alkaloids in *Datura*.

*Datura* species start producing tropane alkaloids as early as six days after germination [72], or when the germinated roots reach approximately 3 mm long [97]. An excellent, in-depth review of the biosynthesis of tropane alkaloids was provided by Kohnen-Johannsen and Kayser [12], and an overall scheme is shown in Figure 6. The roots are believed to be the principal site of tropane alkaloid biosynthesis, and the precursors of the tropane (and pyrrolidine) alkaloids are ultimately the amino acids arginine, glutamine, and ornithine.

These amino acids can be converted to putrescine (**14**, Figure 6) through the action of several enzymes. The enzyme putrescine methyltransferase (PMT) [62,98] *N*-monomethylates putrescine to yield **15**, which is then oxidatively deaminated by methylputrescine oxidase (MPO). The resultant unstable amino aldehyde (**16**) spontaneously cyclizes into *N*-methylpyrrolinium ion (**17**). This pyrrolinium species is electrophilic and can react with a variety of nucleophiles. Pyrrolidine, ecgonine, and tropinone-derived alkaloids are all derived from this intermediate. To form tropinone-derived alkaloids (which represent the majority of the tropane alkaloids present in *Datura* species), this intermediate condenses with two molecules of malonyl-Coenzyme A (CoA) to yield the pyrrolidine oxobutanoic acid **18**. The enzyme that catalyzes this condensation in *Atropa belladonna* roots was recently revealed to be a polyketide synthase (*AbPYKS*) [99], and similar enzyme activity probably serves the same function in *Datura*. This intermediate was identified as a tropane alkaloid precursor in *D. stramonium* root cultures, as ^13^C from a labeled ethyl ester precursor of **18** that was fed to the cultures was found incorporated into (*S*)-**1** and other tropanes [63]. Similar results were observed in feeding experiments using hydroponically-grown *D. inoxia* [100].

**Figure 7:**
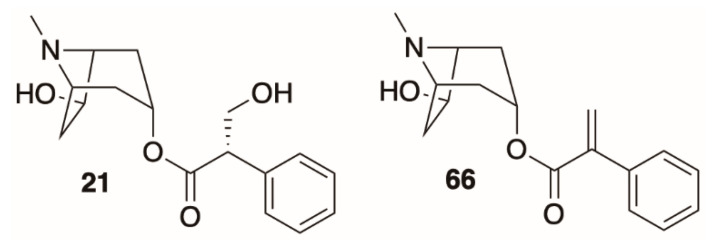
3,7-Disubstituted tropanes found in *Datura*.

The intermediate **18** is then cyclized, with concomitant oxidation and decarboxylation, to afford tropinone (**19**). In *A. belladonna*, the enzyme that catalyzes this cyclization is a cytochrome P450 (called *AbCYP82M3*) [99]; no analogous enzyme has been isolated or purified from the genus *Datura* to date. Tropinone (**19**) has several fates: it can be converted to tropine (α-**10**, the 3α-anomer) by tropinone reductase I (TRI), or pseudotropine (β-**10**, 3β-anomer) by tropinone reductase II (TRII); both of these enzymes have been isolated from *D. stramonium* root cultures [101]. Tropine (α-**10**) gives rise to many acyltropines, including hyoscyamine (*S*)-**1**), littorine (**11**), datumetine (**8**), and more functionalized compounds such as scopolamine (**2**), meteloidine (α-**20**, SI Table S3), anisodamine (**21**, Figure 7), and anisodine (**22**) (SI Table S4). Pseudotropine also yields aliphatic esters such as tigloidine (β-**23**), and is the precursor of the polyhydroxylated calystegines. More information about the biosynthesis of more specific alkaloids is provided under individual subheadings below.

### 4.3. Monosubstituted Tropanes

All reported monosubstituted tropanes, for the exception of 7-hydroxytropane (See SI Table S1) are substituted in the 3-position, and include **19**, α-**10**, β-**10**, and esters derived from the latter two compounds (**1**, **8**, **11**, **23–39**). Numerous monoacylated tropanes have been identified in *Datura* species (mostly by GC-MS), from both intact plant parts and transformed root cultures. Most of these alkaloids have α stereochemistry at the 3-position, and are derived from α-**10**, but a significant number of β-**10**-derived alkaloids, such as tigloidine (β-**23**), have also been detected. Unfortunately, in many cases, the stereochemistry at the 3 position has not been reported; in Table S1 these instances are cited under “α or unspecified”.

Robins [102] reported that different *Datura* acyltransferases act depending on whether the substrate is α-**10** or β-**10**. Individual acyltransferases also may have promiscuous substrate specificity. For example, tigloyl pseudotropine acyltransferase isolated from *D. stramonium* root cultures has the highest activity with tigloyl-CoA, but can also use acetyl, propionyl, isobutyroyl, and senecioyl-CoAs (among other aliphatic substrates) to acylate β-**10**, suggesting that the abundance of a given acyltropane may in part arise from competition between different substrates in the plant’s acyl-CoA (or other acyl donor) pool [102,103]. The aliphatic CoAs utilized by these enzymes are derived from short-chain (C_2_-C_5_) carboxylic acids. Generally, aliphatic acyl groups containing more than five carbons have not been observed in *Datura* alkaloids. Most short-chain carboxylic acids are derived from amino acids. 2-Methylbutanoyl and tigloyl groups (both very common in *Datura* tropanes) are synthesized from isoleucine [49,104], whereas isobutyroyl and 3-methylbutanoyl (isovaleroyl) groups are derived from valine and leucine, respectively [105,106].

Most of *Datura*’s aromatic monoacyl tropanes (SI Table S1) contain phenylacetyl (**30**), 2-phenylpropionyl (**34**), phenyllactoyl (littorine, **11**), or tropic acid moieties [(*S*)-**1** and its derivatives]. One interesting exception is the hydroxylated phenylacetate derivative homatropine (**31**), which is a synthetic drug. Temerdashev [41] detected this alkaloid in *D. metel* extracts using LC-MS, but did not elaborate on how it was identified as homatropine. Another exception is datumetine (**8**), a 4-methoxybenzoyl ester isolated from *D. metel* [45] and characterized by NMR. This alkaloid is unique because benzoyl and substituted benzoyl esters are not found in *Datura*, let alone in most of the Solanaceae; these esters are more common in the Erythroxylaceae (e.g., cocaine) and Convolvulaceae [96].

All phenyllactoyl and tropic acid moieties in *Datura* alkaloids are ultimately derived from phenylalanine, via reduction of phenylpyruvate. In *A. belladonna* roots, an aromatic amino acid aminotransferase (termed *Ab-ArAT*) is found co-expressed with tropane biosynthesis genes; it is anticipated that a similar “ArAT enzyme” produces phenylpyruvate in *Datura* [107]. Post-phenylpyruvate reduction, phenyllactate is then esterified to tropine to form littorine (**11**) by littorine synthase. This enzyme is a specific acyltransferase that uses phenyllactoylglucose as the acyldonor [108]. Hyoscyamine ((*S*)-**1**) is present in all *Datura* species (and root cultures derived from these species), and is frequently one of the most abundant alkaloids, although its abundance depends on the species, organ and ontogenetic stage examined. Hyoscyamine (*S*)-**1** is sometimes interchangeably referred to as “atropine” in the literature, although this is a misnomer, as “atropine” (**1**) refers to pharmaceutical preparations of racemic hyoscyamine, whereas the tropic acid moiety is reported to be almost exclusively (*S*)-(–)- in *Datura stramonium* [44,87].

The tropic acid moiety is derived from the phenyllactate of **11**, isomerized by a P450 enzyme called littorine mutase. The kinetics of (*S*)-**1** formation (i.e., isomerization of the phenyllactate of **11**) in *Datura* (specifically, in *D. inoxia* root cultures) are reported to be slow (around half the rate of the synthesis of **11** itself), suggesting that this may be the rate-limiting step in the synthesis of (*S*)-**1** [109]. The mechanism of this isomerization is still disputed, but computational studies suggest that a P450-mediated concerted cationic rearrangement to yield hyoscyamine aldehyde is a low-energy process [110]. Indeed, masses consistent with hyoscyamine aldehyde (which is then presumably reduced to **11**) have been observed by LADI-MS in *D. leichhardtii* seeds [89].

The tropic acid moiety itself is also further elaborated in *Datura* (SI Table S1); it is hydroxylated at the α-position (alkaloid **35**) in *D. stramonium*, *D. wrightii*, *D. ceratocaula*, and *D. inoxia*, methylated (**36**) in *D. inoxia* and *D. stramonium*, and acetylated (**37**) in most species. One unique hyoscyamine derivative is the *O*-formyl ester of hyoscyamine (α-**38**) observed in *D. ceratocaula*; GC-MS revealed a M^+^ ion of *m/z* 317, which showed a characteristic loss of HCOO• (45 Da) to yield protonated apoatropine (*m/z* 272) [73]. This formyl ester (to date) is unreported in other species. The dehydration product of hyoscyamine (or elimination product of other hyoscyamine derivatives), called apohyoscyamine or apoatropine (**39,** SI Table S1)) has also been commonly observed in GC-MS profiles of *Datura* species. There is debate as to whether this alkaloid legitimately exists in nature, or is an artifact produced by thermal dehydration of (*S*)-**1** (or a derivative) in heated injectors during GC-MS. For example, Dupraz states that **39** was not detected by HPLC analysis of extracts of *D. quercifolia* (root cultures and intact plants), but **39** was detected when the same extracts were analyzed by GC-MS, suggesting it is artifactual [67]. By contrast, in Temerdashev’s 2012 study, **39** was observed in LC-MS traces of *D. metel* extract, suggesting that either it is formed in planta or during extraction (rather than during analysis) [41].

### 4.4. Disubstituted Tropanes

Many *Datura* tropane alkaloids are hydroxylated (or acylated) or epoxidized on the tropane core. Most documented tropane hydroxylation (and epoxidation) occurs in positions 6 and 7, exclusively in the *exo* (β) position, and is catalyzed by the enzyme Hyoscyamine 6β-Hydroxylase (H6H). This enzyme, a nonheme, iron-containing 2-oxoglutarate-dependent dioxygenase, has been cloned from *D. inoxia*, and recombinant H6H from *D. metel* has been expressed in *E. coli* [111,112]. In both cases, the isolated H6H enzyme could also convert (*S*)-**1** to scopolamine (**2**), showing that it can construct epoxytropanes as well. The *D. metel* enzyme also could produce both 6-hydroxyhyoscyamine (**40**, SI Table S2) in addition to scopolamine, but the epoxidase activity (discussed in more detail below) was much lower than the hydroxylase activity.

Table S2 summarizes known *Datura* disubstituted tropane alkaloids (**40–65**). Like their monosubstituted counterparts, disubstituted tropanes are found in all *Datura* species (and in root cultures derived from those species). In addition to 3,6-dihydroxytropane (**41**), these alkaloids contain either one ester and one hydroxyl group or two esters. Interestingly, other than 3,6-diacetoxy (**42**) and -ditigloyloxy (α/β-**43**) tropanes, duplicate ester groups (e.g., diisobutryloxy, diisovaleroyl, or dipropionyloxy) have not been reported, suggestive of enzymatic recognition. Most disubstituted tropanes are aliphatic, although hydroxylated and acylated derivatives of phenylacetoxytropane, apohyoscyamine, and hyoscyamine (such as **40**, **55–57**, **62–65**) have also been found in several species.

Although almost all disubstituted *Datura* tropanes are 3,6-disubstituted, several alkaloids exhibit the rare 3,7-disubstitution pattern. 7-Hydroxyhyoscyamine (**21**, Figure 7), also known as anisodamine, is distinguishable from 6-hydroxyhyoscyamine (**40**) by its GC retention index on an achiral column, as it is a diastereomer of **40**, rather than an enantiomer. This alkaloid has also been detected in *D. inoxia* [113,114], *D. ceratocaula* [73], and *D. stramonium* [72,115]. 7-Hydroxyapoatropine (**66**) [72], was also observed in *D. stramonium*. Two unique disubstituted tropane alkaloids are 3-formyl-6-tigloyloxy tropane (**54**, from *D. stramonium*), which is the only reported alkaloid of this type containing a formate ester, and 3-hydroxy-6-(2-methylbutanoyloxy)-tropane (α-**9**, SI Table S2), which was isolated from *D. ceratocaula*. Beresford and Wooley [49] determined the stereochemistry of the 2-methylbutanoate side chain to be (+)-(*S*). The stereochemistry of 2-methylbutanoate side chains has not been determined or reported in other instances, although it is plausible that they are also (+)-(*S*), if they are derived from *L*-isoleucine as is proposed (vide supra).

### 4.5. Trisubstituted and Epoxytropanes

More elaborate trisubstituted and epoxidized tropane alkaloids have also been found in *Datura*, suggesting that the enzymes that perform hydroxylation, epoxidation, and acylation have evolved to recognize a variety of substrates. 3,6,7-Trisubstituted tropanes (**7**, **20**, **67–77**) are in summarized in Table S3; 3-substituted 6,7-epoxytropanes are likewise in Table S4. All trisubstituted tropanes detected in or isolated from *Datura* species contain either two acyl groups and one hydroxyl group (as in the very abundant and common 3,6-ditigloyloxy-7-hydroxytropane, α-**72**), or two hydroxyl groups and one acyl group, such as 3-tigloyloxy-6,7-hydroxytropane (α-**21**) which was first isolated from *D. meteloides* and thus given the common name meteloidine.

As with the disubstituted tropanes, substituents at positions 6 and 7 have been exclusively assigned β-stereochemistry, whereas position 3 can be α or β, and the only substituents found to repeat in the same compound are tigloyl (as in α/β-**72**) and hydroxyl (as in **20**, **67**, and **75**, SI Table S3). Interestingly, 3,6,7-triacylated tropanes have never been reported in the genus *Datura* [4,96]. In fact, they are not reported to exist in the *Solanaceae* at all; the only representative of this class thus far has been found in *Erythroxylum zambesiacum* (Erythroxylaceae) [116]. Additionally, only two unique trisubstituted tropanes from *Datura* are derived from (*S*)-**1**. Vitale and co-workers [37] detected (by GC/MS) an alkaloid they proposed to be 7-hydroxy-6-propenyloxy-3-tropoyloxytropane (α-**77,** SI Table S3) in the seeds of *D. ferox*. The structure was proposed on the basis of a) loss of formaldehyde by McLafferty rearrangement (consistent with tropic acid derivatives), and b) loss of 72 Da corresponding to the propenyloxy group. Welegergs et al. [48] isolated 3-(3’-methoxytropoyloxy)-6-tigloyloxy-7-hydroxytropane (β-**7**) and assigned its structure by NMR. The 3-position stereochemistry for this alkaloid is drawn as β, although the report does not specify how this assignment was confirmed, nor is the stereochemistry of the tropic acid side chain explicitly specified.

The most abundant epoxytropane in the genus *Datura* is scopolamine (**2**), which is found in all species and hairy root cultures derived therefrom. Most epoxytropanes (SI Table S4) are aromatic, derived from tropic (**2**, **22**, **81**), apotropic (**82**), 3-phenylpropanoic (**83**), or phenylacetic acid esters (**84**), although 3-tigloyl-, 3-hydroxy-(scopine), and 3-acetyl-6,7-epoxytropanes (**80**, **78**, and **79**), respectively) have also been observed in *D. stramonium* and/or *D. inoxia*. As described above, epoxidation, like hydroxylation, is catalyzed by H6H. Epoxidation can occur by dehydrogenation of hydroxytropanes, and in vitro experiments have shown that 6,7-dehydrotropanes are also recognized as epoxidation substrates [12]. Ushimaru and colleagues [117] synthesized a series of tropane analogues as mechanistic probe substrates for H6H. The use of these probes revealed that tropane epoxidation does *not* occur via a dihydroxytropane intermediate, but by radical abstraction of a β-hydrogen one carbon removed from the hydroxyl group. The substrate configuration required for epoxidation is rather strict—the configuration between the β-hydrogen and hydroxyl group must be *syn*-periplanar for epoxidation to occur. If there is no hydroxyl group (i.e., monosubstituted tropane), hydroxylation will occur instead, and if the hydroxyl and β-hydrogen cannot adopt a *syn*-periplanar configuration, dihydroxylation predominates over epoxidation [117].

### 4.6. Dehydrotropanes, Nortropanes, and Calystegines

**Figure 8:**
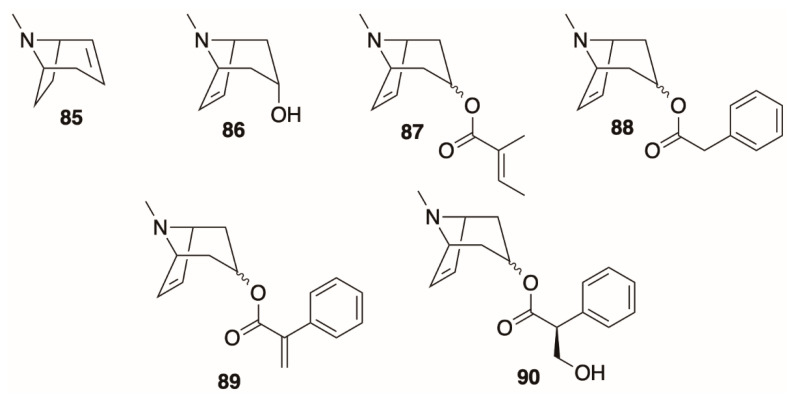
Dehydrotropanes identified in *Datura* species.

A rarer class of tropanes are those with a double bond in the tropane nucleus (dehydrotropanes). The first dehydrotropane discovered in *Datura* was 2,3-dehydrotropane (**85**, Figure 8), which was detected by GC-MS in diploid and tetraploid hairy root cultures derived from *D. stramonium* [118]. The remaining known alkaloids of this class (**86–90**) are 6,7-dehydrotropanes, which were found in *D. stramonium* (**87**), *D. inoxia* (**88**), or both (**85**, **86**, **89**, **90**) by El Bazaoui and co-workers [64,113] Virtually nothing is known (or even proposed) about the biosynthetic origin these alkaloids, although, like apotropoyl derivatives, it is considered that they could be thermal artifacts formed by dehydration or elimination of hydroxyl or ester groups during GC-MS. In LC-MS studies from our own laboratory, however, we have detected dehydrotropanes in neutral water/methanol extracts of both *D. stramonium* and *D. metel* parts, suggesting that some of these alkaloids are formed *in planta*; De-la-Cruz’s (2020) LC-MS observations also support this [75].

*Nortropanes* (Table S5) are tropane alkaloids lacking the methyl group on the tertiary amine. The enzyme responsible for tropane *N-*demethylation has not been isolated from *Datura* or characterized biochemically, although it may act in an oxidative fashion similar to the enzyme that demethylates nicotine [119]. All nortropanes (**91–104**) detected in *Datura*, for the exception of 3-tigloyloxynortropane (**92**) and 3-acetoxynortropane (**91**), are aromatic, and many are epoxy or apotropyl derivatives. It is worth noting that although various other thermal reactions are possible in the GC-MS, thermal tropane demethylation is not considered likely, so observations of presence or absence of nortropanes can be considered a true metabolic difference and not artifactual. In a 2006 chemotaxonomic study by Doncheva et al. [40] the distribution of nortropanes was heavily dependent on the part of the plant studied. Nortropanes were found only in the leaves of *Datura* species; they were not detected in the roots of multiple varieties or accessions of *D. stramonium*, *D. ferox*, *D. inoxia*, *D. wrightii*, *D. leichhardtii*, *D. metel*, or *D. ceratocaula.*

**Figure 9:**
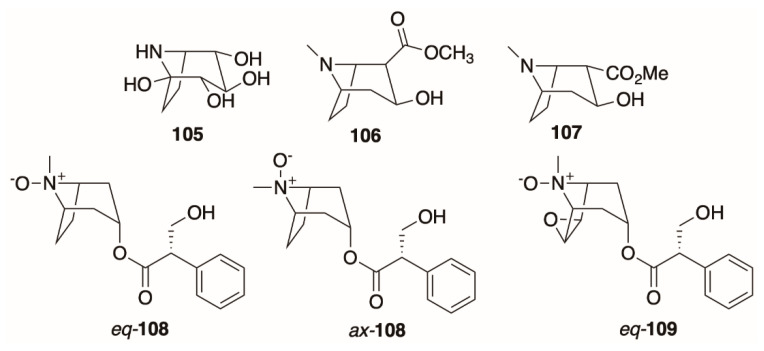
Calystegine, ecgonine alkaloids, and tropane *N*-oxides found in *Datura* species. Note that the “pseudo-” nomenclature for ecgonine alkaloids refers to the stereochemistry of the 2-carboxylate, *not* the 3-hydroxyl.

*Calystegines* are a special class of norpseudotropanes [119] sometimes referred to as “sugar mimic” alkaloids because of their extensive hydroxylation and ability to inhibit various glycosidases [54]. Calystegines are challenging to detect because they have high aqueous solubility that impairs liquid-liquid extraction into organic solvents, do not run well on GC without prior derivatization, lack useful UV chromophores, and do not stain with Dragendorff reagents. Although the genes required to produce calystegine precursors (pseudotropine, β-**10**) are present in *Datura*, reports of these alkaloids are rare for the genus. Nash [54] reported calystegine B2 (**105**, Figure 9) in *D. wrightii* leaves, whereas Drager and colleagues [120] reported the presence of calystegines in *D. stramonium* leaves and roots, although their contents were too low (<0.5 µg/g tissue) to quantify reliably, and they were not detected in cultured roots of *D. stramonium* [121].

### 4.7. Ecgonine Derivatives, Tropane N-Oxides, and Cyclic and Dimeric Tropane Alkaloids

The ecgonine alkaloids (2-carboxy-3-hydroxytropanes) are the class of tropane derivatives that includes cocaine and its derivatives and precursors. These alkaloids, which share similar pyrrolidine-based biosynthetic origin to tropinone-derived alkaloids [12] are more common in the Erythroxylaceae than the Solanaceae [4]. There are several exceptions: methylecgonine (**106**, Figure 9) was observed by Berkov in the roots and stems of *D. ceratocaula*, with the stems having a slightly higher content as assessed by GC-MS (% of total ion chromatogram) [76]. Similarly, **106** was reported in low concentrations in *D. stramonium* stems and roots, and *D. inoxia* roots, stems, leaves, and flowers—accessions of both species were harvested from Northwestern Morocco [64,113]. Additionally, **106** was found in both diploid and tetraploid hairy root cultures derived from *D. stramonium* [119]. Methylpseudoecgonine (**107**) was also discovered in *D. stramonium* and it was identified as such by comparison of the natural alkaloid with all four synthesized isomers of methylecgonine by GC-MS [122]. This alkaloid was also found in *D. stramonium, D. stramonium* var. *tatula,* and *D. inoxia* harvested from Algeria [115].

Although unusual, the tropane nitrogen can become oxidized, yielding a tropane *N*-oxide (such as hyoscyamine *N*-oxide). Both an axial (*ax*-**108**) and an equatorial (*eq-***108**) isomer of this alkaloid exist (Figure 9) and are separable; both have been isolated from *D. stramonium* root, stem, leaf, flower, and pericarp; the equatorial isomer of scopolamine *N*-oxide (*eq-***109)** was isolated from the same plant and parts [123]. Tropane *N*-oxides are problematic to detect, isolate, and characterize because of their poor solubility in common extraction solvents, such as ether, and their propensity to thermally deoxygenate and demethylate during GC-MS, which makes this a poor detection method [68]. As such, their structures were assigned by NMR. Interestingly, Vitale [37] reported that no tropane *N*-oxides were detected in *D. ferox* seeds; there is little comment in the literature regarding the presence of these derivatives in other *Datura* species.

**Figure 10:**
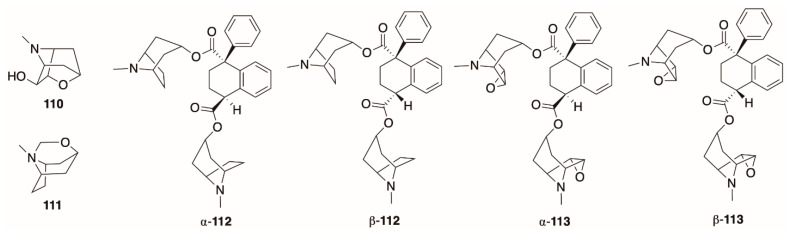
Cyclic and dimeric tropane alkaloids from *Datura*.

The cyclic tropane (+)-scopoline (**110**, Figure 10), also known as oscine, was first isolated from the genus *Datura* by Heusner in 1954 [124]. Since then, it has been observed in *D. stramonium* [68,72,74], *D. inoxia* [113,114], and *D. wrightii* [40]. A second cyclic tropane, cyclotropine (**111**), was observed in *D. stramonium* and *D. inoxia* [113,114]. Ionkova and Jenett-Siems propose that these compounds may be artificially produced during the isolation procedure (arising from degradation of epoxytropanes followed by intramolecular nucleophilic attack of the 3-hydroxyl on the epoxide), or during GC-MS, although both of these alkaloids were also observed by LC-MS during De-la-Cruz’s profiling of *D. stramonium* leaves [61,75,122].

*D. stramonium* contains the dimeric alkaloid belladonnine (α/β-**112**, Figure 10), which consists of two tropine (α-**10**) moieties esterified to a tetrahydronaphthalene diacid known as isatropic acid. Aripova and Yunusov propose that isotropic acid may be formed by photodimerization of atropic acid (dehydrated tropic acid, the hydrolysis product of apohyoscyamine (**39**, SI Table S1)) [125]. Prolonged heating of hyoscyamine can also produce this dimer. An analogous ester of scopine (**78**, SI Table S4) and isatropic acid, called scopadonnine (α/β-**113**) was isolated from *D. inoxia* seeds. This alkaloid, like **112**, is naturally isolated as a mixture of α-(*anti*) and β-(*syn*) isomers, and the structure of β-scopadonnine (α/β-**113)** was unambiguously determined by *N*-methylation with methyl iodide, recrystallization of the di-quaternary salt, and X-ray crystallography [126].

## 5. Alkaloids of the Genus *Datura*—Non-Tropane Alkaloids

Thus far, this review has classified compounds using the classical definition of alkaloids—natural products containing a basic (and often heterocyclic) nitrogen, or those compounds biogenetically related to basic alkaloids. In addition to tropanes, there are many other types of nitrogenous compounds in *Datura* species, such as hydroxycinnamic amides, amino acids, diketopiperazines, and their derivatives; those are not considered alkaloids for the purposes of this discussion, although other studies have used the term “alkaloid” more broadly [36,77,127].

### 5.1. Pyrrolidines

**Figure 11:**
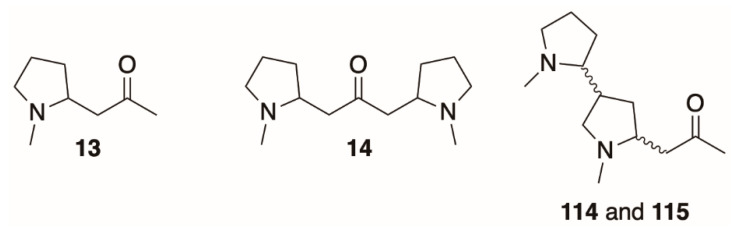
*N*-methylpyrrolinium ion (**17**)-derived pyrrolidine alkaloids.

Pyrrolidine alkaloids (Figure 11) are products of the reaction of electrophilic *N*-methylpyrrolinium ion (**17**, Figure 6) with various nucleophiles. The most common pyrrolidine alkaloid in the genus *Datura* is hygrine (**12**), which results from the condensation of **17** and acetoacetate (derived from two molecules of acetyl-CoA) [19]. Although originally believed to be such, this alkaloid is not a tropane precursor, but rather a by-product of the tropane biosynthetic pathway [63]. Two enantiomers of **12** are possible, although the literature does not explicitly elaborate on which are found in *Datura* (it is drawn as racemic). *D. inoxia* and *D. metel* var. *fastuosa* roots, but not leaves, are reported to contain small amounts of hygrine [40]. This alkaloid was also detected in the seeds of *D. ferox* from Argentina [37], *D. stramonium* from Egypt and Mexico [22,75], in *D. leichhardtii* seeds [87], and in root cultures derived from *D. stramonium*, *D. inoxia*, *D. metel* var *fastuosa*, *D. quercifolia*, *D. ferox*, and *D. wrightii*) [61,128].

Cuscohygrine (**13**) results from condensation of oxobutanoate ester **18** (Figure 6) with **17**, followed by decarboxylation, [99] although labeling studies suggest it can also be formed from **12** [63]. Although it can exist as two diastereomers (two pairs of enantiomers), no references are made to the specific isomer(s) found in *Datura*, although both isomers of **12** and **18** convert to **13** [63,129]. This alkaloid has been found in *D. inoxia* [39,114], *D. stramonium* [68,75] *D. metel* var. *fastuosa*, *D. leichhardtii*, and *D. ferox* [40]. It is found in only low amounts in *D. ceratocaula* [73], but it is reported to be the most abundant alkaloid in *D. discolor* roots (around 20% of the total alkaloid content) [130]. Cuscohygrine (**13**) is also found in root cultures derived from *D. stramonium*, *D. ferox*, *D. wrightii*, *and D. inoxia*, and is 10% of the total alkaloid content in the last species [118,128].

Two other pyrrolidine alkaloids, called *N*-methylpyrrolidinylhygrines A and B (**114** and **115**) were detected by GC-MS in 1987 in *D. inoxia* roots [47]. On the basis of the fragment ions in mass spectra, the structures were only putative, but it was proposed that there are two isomers. Two isomers were also found in *D. inoxia* root cultures [61] and later in *D. ceratocaula* [73]. It is almost certain that *Datura* species contain more undiscovered pyrrolidine alkaloids. In Witte’s GC study [39] of *D. inoxia* roots, nitrogen-specific detection suggested that one alkaloid (*m/z* 414, M^+^) contained more than one nitrogen, and yielded EI-MS fragments of *m/z* 124 and 84, suggesting that this alkaloid contained both pyrrolidine and tropane moieties. This is unusual, as tropane alkaloids containing additional nitrogen atoms (excluding dimeric tropanes) have not been reported in the Solanaceae. “Alkaloid 414” has also been recognized in the transformed roots of *Brugmansia* and intact plants and root cultures of *Hyoscyamus* species but no structure was proposed [128,131].

### 5.2. Indoles and Beta-Carbolines

**Figure 12:**
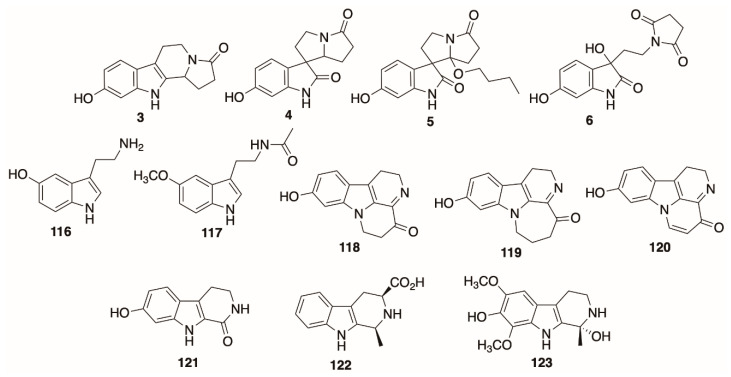
Indole and *beta*-carboline alkaloids found in *D. stramonium* and *D. metel*.

The second major non-tropane class of alkaloids found in the genus *Datura* are those based on indole or oxindole cores (Figure 12). In 2020, four racemic indole alkaloids (daturametelindoles **3–6**) were isolated from an ethanolic extract of *D. metel* seeds by Liu et al. [47] who used 2D-NMR, chiral LC, polarimetry, and circular dichroism spectra to resolve structures and stereochemistry. Interestingly, the *n*-butyl ether of compound **5**, to the outsider, appears to be a potential artifact of isolation, as *n*-butanol was used during fractionation, but a 2019 study of rodent metabolism of *D. metel* metabolites detected **5** in an aqueous extract of the seeds (where no *n*-butanol was used), suggesting that the *n*-butyl group occurs naturally in the plant [77].

While daturametelindoles B-D are oxindoles, daturametelindole A (**3**) appears to be hydroxytryptamine-derived. In that context, it is interesting that Murch and colleagues (2009) found the hydroxytryptamine serotonin (**116**) along with melatonin (**117**) in the flowers and buds of *D. metel* var. *fastuosa*. Interestingly, the compounds were most abundant in immature flower buds, and levels decreased as the buds matured and flowered. Cold stress also significantly increased melatonin concentration in the buds, suggesting that it may be part of a protective or stress response [132].

*Beta*-carbolines (similar in structure to the *Peganum harmala* alkaloids) have also been observed in *Datura*. In 1981, Maier and co-workers sliced *D. stramonium* (all varieties) seeds and noted that the cut surface had a distinctive blue-green fluorescence under UV light. This fluorescence was attributed to two compounds later isolated by a combination of column and preparative thin-layer chromatography—the *beta*-carbolines fluorodaturatin (**118**) and homofluorodaturatin (**119**) [133]. A 1,2-dehydro analogue (**120**) was subsequently reported in 1984 [134]. Similar *beta-*carboline structures are reported in *D. metel*. In Hui’s metabolomics study of this plant, alkaloids **121** and **122** were noted, but they appear to be in low abundance in the reported chromatograms [36]. Okwu and Igara also isolated what they proposed to be the antibacterial *beta*-carboline alkaloid **123** from *D. metel* leaves from a Nigerian plant accession, although the authors did not use any additional NMR techniques (such as COSY, TOCSY, or HMBC) to confirm connectivity, so their structure should be treated as putative [135].

### 5.3. Miscellaneous Alkaloids

**Figure 13:**
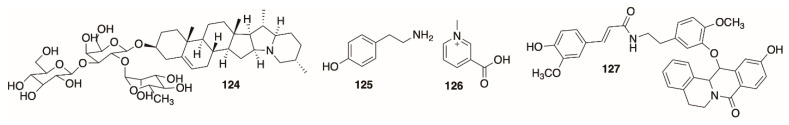
Miscellaneous alkaloids identified in *Datura*.

Kariñho-Betancourt [76] reports that the toxic glycoalkaloid solanine (**124**, Figure 13) is present in the leaves of most *Datura* species, albeit as a minor alkaloid whose concentration changes very little with age. Solanine is very common in the Solanaceae and is also found in tomatoes, potatoes, and eggplant, among other species. In Witte’s [39] GC study, tyramine (**125**) was found in *D. inoxia* leaves and flowers. Similarly, a joint Japanese-Bangladeshi group [136] also identified tyramine as in *D. stramonium* leaves while performing bioassay-guided fractionation in search for compounds that would induce apoptosis in cancer cells. The presence of free tyramine in these plants is unsurprising, as it is commonly incorporated into hydroxycinammic acid amides and similar compounds. The same group [136] identified the pyridine trigonelline (**126**) in the same screen; although, like tyramine, this compound is found across many genera and plant families and is not unique to or characteristic of *Datura*. Hui et al. [36] reported that the tetrahydroisoquinoline derivative chenoalbicin (**127**) is present in many parts of *D. metel* but is the highest in the seeds. This compound was identified by matches with its exact mass, retention time, and two MRM transitions from *m/z* 607>353 and *m/z* 607>277 matching a standard.

## 6. Factors Affecting Alkaloid Content and Composition 

As described in the previous sections, the most obvious factor influencing alkaloid composition within the genus *Datura* seems to be genotype and tissue type [34,35,40,74,128,137,138]. Nonetheless, a multitude of other variables can affect alkaloid identity and diversity, total alkaloid content, and ratios between different alkaloid levels in *Datura*; those variables are briefly summarized in the following subsections.

### 6.1. Location within the Plant

Although all parts of all *Datura* plants contain alkaloids, the roots are considered the primary site of alkaloid biosynthesis, and alkaloids are then trafficked throughout the plant. This was largely confirmed by the elegant grafting studies performed by James [53], where *D. inoxia* and *A. belladonna* roots and aerial parts (species which contain very different alkaloid profiles) were grafted onto each other. The resulting aerial tissues contained the alkaloid composition and ratio of the species used as the rootstock.

As roots are the ultimate origin of most alkaloids, their diversity and concentrations are usually highest in these parts. For example, Evans and Somanabandhu [130] found that the alkaloid content in the roots of *D. discolor* was 0.31% of the dry weight, as compared to only 0.17% in the aerial parts. Doncheva [40] also reported that the roots of various *Datura* species exhibited a greater “variety of alkaloids” than the leaves, although some leaf-specific alkaloids (such as nortropanes) were also found. Similarly, the aerial parts of *D. inoxia* contain mostly (*S*)-**1** and **2**, whereas the alkaloid content of the roots was much more diverse and included various pyrrolidine alkaloids in addition to tropanes (*vide supra*) [39].

In contrast to other studies, Miraldi et al. [65] described that roots of adult *D. stramonium* originating in Italy generally do not contain appreciable amounts of alkaloids, but also propose that alkaloids may be synthesized in the roots of younger plants and then elaborated in the aerial parts after transport, a hypothesis which was also supported by Berkov [72] and others.

There is considerable variation in alkaloid content between the different aerial parts of individual plants as well: (*S*)-**1** and **2** contents were found to vary significantly between the aerial parts of adult *D. stramonium* plants, with the highest concentration of these alkaloids being found in the seeds, and the lowest in the fruit pericarp and stem [65]. A similarly high concentration of scopolamine was observed in *D. metel* seeds of four varieties, but flowers and leaves of the same plants contained lower amounts [24]. Witte [39] describes similar differences between the aerial parts of *D. inoxia*. Although tyramine (**125**, Figure 13) was present in both flowers and leaves, it was one of the three major constituents of the flowers, whereas the major components of the stems are (*S*)-**1**, **2**, and α-**20** (meteloidine, SI Table S3).

### 6.2. Plant Age

One of the earliest studies that quantified alkaloid content with respect to age was that of Fuller and Gibson [51] who spectrophotometically measured (*S*)-**1** concentration in *D. stramonium* var. *tatula* plants at different weeks of growth. They found that (*S*)-**1** concentration increased in both roots and leaves until around 10 weeks of age, after which it appeared to plateau. There are many contemporary (and conflicting reports) concerning the relationship between plant age and alkaloid content. Kariñho-Betancourt [76] reported that tropane alkaloid concentration is generally higher during the reproductive stages of all species of *Datura* plants studied (as assessed by LC-MS), whereas Miraldi [65] stated that young *D. stramonium* stems and leaves contained considerably higher amounts of (*S*)-**1** and **2** than the analogous parts of adult plants. A 1989 study [35], on the other hand, revealed that the levels of these alkaloids actually fluctuate in different plant parts over time, suggesting that alkaloid levels reflect a complex interplay between synthesis, transport, and degradation. Jakabová et al. [139] surveyed (*S*)-**1** and **2** contents in *D. metel*, *D. stramonium*, and *D. inoxia*, and compared those plants harvested in Hungary in either the summer and autumn, and the autumn samples (older plants) contained considerably lower alkaloid content than the summer plant samples. It is possible that in this case, seasonal changes also influence alkaloid content, in addition to ontogenetic stage (vide infra).

The exact composition and type of alkaloids found changes with plant age as well: in the roots of yellowed, senile *D. stramonium* plants harvested in autumn, only five alkaloids were detected by GC-MS, suggesting very slow tropane alkaloid synthesis (or shunting of alkaloids into the fruits), whereas forty-two different tropane alkaloids were detected in the roots while the fruits were maturing [72]. This same study also found that in the aerial parts of *D. stramonium*, the percentage of 6,7-epoxytropanes (of all total alkaloids) decreased with age, whereas the percentage of monosubstituted tropane alkaloids increased correspondingly, which suggests possible degradation/recycling or altered alkaloid trafficking as the plant ages.

### 6.3. Ploidy of Plants

The alkaloid content can also be influenced by the chromosome copy number (ploidy) of the plant. Berkov and Philipov [140] collected natural diploid *D. stramonium* in Bulgaria and artificially created autotetraploid plants by treatment of seedlings with colchicine. While the overall alkaloid profile was not significantly different, tetraploid plants had a higher concentration of alkaloids in roots and leaves, as well as a higher scopolamine/hyoscyamine ratio, suggesting that tetraploid plants may produce more scopolamine. In hairy root cultures established from both diploid and tetraploid *D. stramonium* plants (via *Agrobacterium* transformation), alkaloid content was measured on the 3rd, 9th, and 15th day of growth. At 15 days of age (the stationary phase of the culture), the diploid hairy root cultures contained more than twice as many different alkaloids as the tetraploid cultures—mostly minor acyltropane derivatives [118]. Similarly, hygrine (**12**) and cuscohygrine (**13**, Figure 11), and methylecgonine (**106**, Figure 10) were found in the diploid, but not the tetraploid cultures. In contrast, the diploid cultures contained less hyoscyamine than the tetraploid cultures at the stationary phase, possibly as a result of its derivatization into other alkaloids. These results suggest that the ploidy of *Datura* may be an important variable to consider for metabolic engineering.

### 6.4. Light and Water Amount

Plant growth conditions can also significantly affect alkaloid production. *D. metel* plants exposed to long days and bright light in Cosson’s 1969 study accumulated more **2** than plants kept in dim light [141]. It was later suggested that flower formation (which happens during lower light periods and can be inhibited by bright light) somehow negatively affects the enzymatic epoxidation of (*S*)-**1** [33]. Demeyer and Dejaegere [142] also observed that high-energy light caused a redistribution of alkaloids (from the leaves and stems to the fruit and seeds) in *D. stramonium* var. *tatula*. A very recent publication, however, reported that while both *D. stramonium* and *D. inoxia* plants kept in low light had altered morphology (lower mass and larger leaf areas), the overall concentrations of **1** and **2** in the leaves did not change among any of the three light conditions tested [35].

An extensive study by Moreno-Pedraza [32] showed that alkaloid content in *D. stramonium* correlated with soil moisture. Trays of growing plants were irrigated with 500, 1000, 1500, and 2000 mL of water every eight days, and alkaloids were then quantified in different tissues by LC-MS. The relationship between alkaloid content and water amount was highly variable. For example, the average littorine (**11**), scopolamine (**2**), and acetylhyoscyamine (**37**) content decreased in all organs when the water volume increased beyond 1500 mL. Hyoscyamine amounts, by contrast, showed a parabolic dependence, being lowest at very low (500 mL) and very high (2000 mL) water conditions. In other cases, the correlation seems to be organ-specific: one isomer of hydroxyhyoscyamine (possibly anisodamine, **21**, Figure 7) peaked in amount at 1500 mL water in leaves and roots, but peaked at 2000 mL water in stems.

### 6.5. Chemical Additives

Alkaloid amounts can also be altered by addition of salts, hormones, growth retardants, elicitors, or other exogenous chemicals. A 1985 study [143] reported that salt stress (addition of 0.1538 M NaCl during growth) enhanced **2** accumulation in the young leaves of *D. inoxia*, although the positive correlation between growth and overall alkaloid content did not change. A later study treated *D. stramonium* var. *tatula* with various mineral solutions. These solutions contained with one dominant anion (nitrate, sulfate, or H_2_PO_4_^-^) and one dominant cation (K^+^, Ca^2+^, or Mg^2+^). Plants treated with nitrate-containing ion pairs had increased hyoscyamine yield (likely as a result of increased nitrogen availability), whereas **2** amounts tended to correlate more with growth stage than the presence or absence of additives [33].

In another study published by the same group, Demeyer et al. [144] treated growing *D. stramonium* with the hormones cytokinin (DMAA) and auxin (IAA). The content of (*S*)-**1** was significantly higher in the leaves of DMAA-treated plants, possibly because of increased metabolism or reduced senescence. In IAA-treated plants, the (*S*)-**1** and **2** contents were lower than the control plants in the whole plant until 20 weeks. In the control plants, alkaloid contents decreased after 20 weeks of age, whereas in the IAA-treated plant, they increased. The authors hypothesized that IAA promotes the pentose phosphate pathway and the glyoxylate cycle, which results in excess glutamate and energy, which are converted into ornithine and, thus, shunted into the putrescine (**14**)/*N*-methylpyrrolinium ion (**17**) pathway (Figure 6).

Gupta and Madan [145] sprayed the aerial parts of *D. metel* var. *fastuosa* with two growth retardants (CCC and B-995), and found that at 20,000 ppm, both agents increased alkaloid content in the plants’ leaves. This may be because those agents behave as gibberellin antagonists; adding a gibberellin (GA3) to hairy root cultures derived from *D. quercifolia* resulted in a concentration-dependent decrease of (*S*)-**1** production [74]. Interestingly, a 2018 study revealed that addition of acetylsalicylic acid and salicylic acid to hairy root cultures obtained from *D. stramonium*, *D. stramonium* var. *tatula*, and *D. inoxia* significantly increased (*S*)-**1** production, peaking at an additive concentration of ~0.1 mM) [115]. The use of these elicitors to increase alkaloid production has been reported in different species, and they are proposed to act at the transcriptional level. In a 2009 study, it was found that the addition of *Agrobacterium rhizogenes* to the nutrient solution of hydroponically grown *D. inoxia* resulted in increased accumulation of (*S*)-**1**, **2**, and enhanced tropane hydroxylation and esterification [146].

### 6.6. Geography, Altitude, Climate, and Season

Geography appears to significantly influence alkaloid composition. This appears intuitive at first glance, as the different climates, altitudes, insect populations, and local microenvironments of different areas could impart different selection pressures on plants. Alexander’s review [6] summarizes the alkaloid contents of *Datura* plants of the same species isolated from different geographical locations, many of which display striking variance in alkaloid composition. For example, Berkov and colleagues [22,114] noticed that both *D. stramonium* and *D. inoxia* (roots, leaves, and seeds) had different patterns of alkaloids when they were grown in Egypt (versus grown in Bulgaria). In *D. inoxia,* scopine (**78**, SI Table S4) was the predominant alkaloid found in Egyptian plant leaves, but (*S*)-**1** was instead predominant in Bulgarian plant leaves. The authors also reported that they did not find ten of the reported alkaloids, including **12** (Figure 11) found in Witte’s *D. inoxia* study, which used plants grown in a greenhouse in Germany [39]. *D. ferox* [147] also showed geographical variation: both **2** and total alkaloid content varied depending on which province of Argentina the samples were collected from. *D. metel* flowers collected in Shandong, Changde, and Guangdong (China) differed in their **21** (Figure 7) **2**, and (*S*)-**1** contents [60], and similar differences were also observed for specimens of *D. metel* collected in different locations in India [52]. Interestingly, the same authors also discovered that plants grown at high altitude locations (Darjeeling and Pachmari) contained considerably higher alkaloid percentages than those grown at lower altitudes (Poona (Pune) and Bombay (Mumbai)).

Similarly, Karnick and Saxena also found a seasonal periodicity to total alkaloid content in *D. metel* grown in India [52]. Cold or rainy weather favored greater total alkaloid percentages in the roots and stems when compared to hot weather. By contrast, rainy weather favored higher alkaloid content in leaves, and alkaloid contents decrease in roots and leaves during the hot seasons as they accumulate in fruits and seeds, although in this case, it is difficult to distinguish which effects result from seasonal changes and which are derived from plant age.

### 6.7. Insect Herbivory

As alkaloids are predicted to be defense compounds, several studies on the relationship between insect herbivory and alkaloid production in *Datura* have been conducted. In Shonle and Bergelson’s [148] study of *D. stramonium* populations planted in Illinois (US), plants preyed upon by herbivores displayed different selection pressures on certain alkaloids. Hyoscyamine (*S*)-**1** came under positive selection (meaning an increase in fitness for those plants), whereas **2** came under negative selection, suggesting that it was costly for the plant to make and conferred some fitness disadvantage, which lead the authors to believe that it may have actually stimulated certain insects to feed. In a study of Mexican *D. stramonium* populations [149], the reverse was observed: herbivores selected for a reduction in (*S*)-**1** concentration, suggesting it was ineffective at deterring herbivory, whereas **2** appeared effective against generalist but not specialized herbivores (as its concentration was increased in plants fed upon by these insects). The authors claim that this lends support to the “evolutionary arms-race” hypothesis, where *Datura* species develop more progressively functionalized alkaloids (such as trisubstituted or epoxytropanes) to circumvent herbivore resistance to simpler compounds (such as (*S*)-**1**). Interestingly, a 2018 study [150] reported that the growth of two species of caterpillars—one that co-evolved with *D. wrightii* and one that didn’t—was unaffected by **2**. This observation suggests that either (a) **2** may serve another purpose altogether—perhaps deterring herbivory or seed predation by mammals, who are affected very strongly by **2**, or (b) it is other *Datura* alkaloids that have anti-insect activity.

Taken together, these studies on the many variables affecting alkaloids provide two valuable conclusions. First, it may be possible to select for the production of certain alkaloids (or increased yields of them) by changing conditions under which plants are grown. Second, because *Datura* alkaloid composition is so sensitive to environmental changes, any study that seeks to assess genome-level (or other biochemical) changes must use plant groups grown and kept under identical and controlled conditions.

## 7. Conclusions and Future Challenges: Stereochemistry, Dereplication, and Observer Bias

As is the case in almost all natural products research, the isolation and identification of metabolites is still the major bottleneck in studying *Datura*. To understand how alkaloids are synthesized, what their ultimate roles in the plant may be, how they relate to substances found in similar plants, or what bioactivities they may have, more of their structures need to be known, and known *unambiguously* beyond the putative annotations that dominate the literature.

First, *Datura* literature is full of proposed uncharacterized alkaloids, described over the years in terms such as “alkaloid A”, “alkaloid 325”, “Alkaloid 414”, and under catch-all headings such as “uncharacterized bases” [22,39,151,152]. What are these alkaloids, and what other yet-undiscovered compounds accompany them? With the full array of tools available to the modern alkaloid hunter, such as preparative LC columns available in a variety of phases, high-resolution mass spectrometry and MS/MS, and advanced and sensitive NMR techniques, these questions are becoming easier to answer. Special attention, however, must be paid to the absolute configuration of compounds, especially tropane alkaloids, where a multitude of different isomers can be possible and confusion regarding the exact stereochemistry of standards has mounted over the years. Techniques such as chemical derivatization, polarimetry, and NMR chiral shift reagents have all been employed successfully, and it is also worth noting that modern and sophisticated optical techniques, such as vibrational circular dichroism (VCD—circular dichroism applied to the infrared and near-infrared light regions) have been applied to tropane alkaloids, just not to those specifically isolated from or unique to *Datura*. For example, Reina et al. [153] used density functional theory calculations to predict probable conformations and VCD spectra of certain disubstituted tropanes and tropane mixtures from *Schizanthus*, and then were able to assign absolute configuration by comparison of experimental spectra to both the calculated and to literature spectra for similar alkaloids. In another study, the same group [154] also added chemical derivatization to this procedure: Compound **40** (SI Table S2) was first converted to **42** by base hydrolysis followed by treatment with acetic anhydride, and then the absolute configuration of **42** was determined as above. The authors found that the VCD spectra of **40** and **42** had several common bands that are proposed to be directly related to the absolute configuration of the tropane nucleus. Humam et al. [155] also utilized a similar “compute-and-compare” procedure with electronic circular dichroism (which uses light in the UV–VIS range) instead of VCD. These avenues should definitely be explored more for determination of absolute configuration of *Datura*-specific alkaloids.

Another prominent challenge in natural products discovery, that runs hand-in-hand with identification, is that of dereplication [156]. How do researchers avoid spending time, money, and resources isolating (or worse yet, chasing the bioactivity of) a compound that turns out to be already known? This is especially a problem for tropane (and other) alkaloids, where isomerism complicates things immensely. For example, a given GC/MS or LC/MS chromatogram may yield multiple peaks that share common molecular and fragment-ion masses. Standards may not be readily available to sort out which isomer is which, synthesizing them all may be cost-prohibitive or labor-intensive, and it may be near-impossible to obtain a large enough amount of a compound for NMR. Even MS/MS, as useful as it is, is not 100% replicable, as spectra can vary between ion source and individual instrument platforms, and can also be affected by ion source conditions, solvents, salt, or matrix effects, so spectral database comparisons have limits in distinguishing compounds [156]. Special attention must therefore be paid to utilizing and improving those dereplication techniques which are most rapid, high-throughput, and invariant.

An additional complication in both identification and dereplication is that even samples that appear “pure” by HPLC may be a mixture of inseparable isomers or other compounds, which is often an unpleasant surprise late in the isolation workflow. One recently developed technique that could aid in situations such as these is ion mobility spectrometry (IMS), which is now integrated into several mass spectrometry platforms. Often coupled to LC, this mass spectrometry technique is a form of post-ionization processing where ions move differently in a “drift tube” based on their shape, charge and size (called a collision cross-section)—effectively gas phase electrophoresis performed in tandem with mass spectrometry in a time-scale consistent with chromatographic separations and mass spectrometry data acquisition. This processing adds an additional degree of separation, and the data is often plotted with the LC chromatogram, yielding a two-dimensional plot of *retention time* versus *drift time*. IMS can aid in separating isomers and revealing new compounds that may not be detected by conventional LC or LC-MS methods [157]. Additionally, IMS provides an additional “data point” in any compound’s given molecular signature, which is valuable in dereplication—two compounds may have identical masses, fragmentation patterns, and retention times, yet differ in their IMS drift times, and are therefore likely different compounds [158]. This method was applied to *beta*-carboline alkaloids from *Peganum harmala,* where more alkaloids were differentiated and identified using the 2D-combined LC-IMS method than by LC or IMS alone [157].

Another emerging issue in natural products research is over-reliance on databases. We live in the Digital Age, and databases such as PubChem, ChemSpider, Metlin, and KEGG are easily used, valuable repositories of information, and software and websites (such as MetFrag or Progenesis QI) are increasingly used to interface with and match empirical mass spectral (and other) data to those in databases. If these results are not examined critically (and within the context of biochemical plausibility) misannotations and misidentifications can (and do) result. Several metabolite profiling studies using database matching [78,79] have presented questionable identifications of components of ipecac, Parkinson’s disease drugs, veterinary antibiotics, bacterial-derived organoboron compounds, streptomycin derivatives, and antidepressant metabolites in *Datura* samples, while failing to identify (*S*)-**1**, **2**, or other commonly reported *Datura* alkaloids. An additional example of output provided by computational analysis open to discussion is found in the study of Moreno-Pedraza [32], where the program MetFamily was used to group *Datura* metabolites into putative classes on the basis of their MS/MS fragmentation, which the authors divide into four classes of “atropine-like” compounds, among others. While the study itself appears scientifically sound with many valid conclusions, some of the species classified by MetFamily as “atropine-like”, such as C_28_H_24_O_5_, C_28_H_38_O_5_ (Withanolide B), and C_25_H_33_FO_4_, are, obviously, *not* tropane alkaloids if these formulae are correct (lacking nitrogen), even if something about their fragmentation may be tropane-like in nature. Further development and refinements of similar computational approaches are encouraged with appropriate testing and validation.

These examples also speak to the difficulty in overcoming observer bias in metabolomics. When do we trust what our software and databases tell us, and when must we rely on intuition? Since many metabolite studies focus explicitly on one class of compounds, the interpretation of our data (especially mass spectral data) is often clouded by human judgment, operating within the framework of our often-limited knowledge across an entire class of compounds. It is often proposed that if a compound exhibits tropane-like mass spectra, it must be a tropane. While assumptions like these may be a good starting point, how many alkaloids have been missed over the years simply because something about their behavior (unusual masses, retention times, mass defects, or fragmentation) does not fit our hyper-focused definition of how an alkaloid should behave? Additionally, if we do not recognize and report a certain compound because it does not meet some threshold for annotation as that compound, is it actually not present, or are we simply not looking for it? These considerations should be taken to encourage deposition of raw analytical data into shared repositories.

Finally, the question of bioactivity must be addressed. Thirty-four percent of new drugs approved between 1981 and 2010 [159] have a direct natural product origin, and *Datura* has been used medicinally for millennia. Nonetheless, many modern studies on the medicinal properties of *Datura* still employ whole-plant part extracts, and comparatively little is known about the active alkaloidal constituents beyond atropine and scopolamine—two alkaloids out of potentially hundreds across the genus, where there is a plethora of interesting bioactivity waiting to be discovered. In addition to their anticholinergic and psychoactive activity, some tropane alkaloids have shown antiviral, antifungal, and antimicrobial properties, while others can reverse multidrug resistance in cancer cells [48,160,161], while the pyrrolidine, indole, and *beta*-carboline alkaloids are also largely unexplored. More screening of isolated *Datura* alkaloids against diverse targets should be performed, and as their structures are elucidated, it will become easier to perform structure-activity relationship studies and design novel semi-synthetic or synthetic alkaloid mimics.

With new advances in analytical instrumentation (and careful study by many researchers) more than one-hundred alkaloids in multiple classes have been described in the genus *Datura*, and successful efforts have been made to determine precise conditions that affect their presence and amounts. Nonetheless, great untapped potential and promise remains in these plants, and the future of alkaloid research remains bright. It is, however, only when we use both critical thought and the full arrays of tools and techniques available to us that we might take our place in the long history of this fascinating and storied genus. After all, who knows what medicines, mysticism, and magic still lie in wait?

## Figures and Tables

**Table 1:** Comparison of tropane and pyrrolidine alkaloid fragment ions obtained by GC-(EI) and LC-(ESI)-MS.

Class	Representative Structure, Where R = Acyl or Hydroxyl	Diagnostic GC-MS (EI) Fragmentation (*m/z,* M^+^)	Diagnostic LC-MS (ESI) Fragmentation (*m/z*)	References
**Monosubstituted tropane**	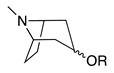	141, 140, 124 (usually base peak), 96, 94 (*N*-methylpyridinium cation), 83, 82 (base peak when no ester at C-3), 42	142 (loss of anhydro-acid), 124 (loss of neutral acid), 93, 91 (loss of methylamine, dehydrogenation), 77	[37,68]
**Disubstituted tropane**	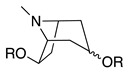	156, 140, 138, 122, 113 (prominent if 3-hydroxyl), 96 (3-hydroxyl), 95, 94 (usually base peak if not 3-hydroxyl), 55, 42	158 (sometimes, dihydroxytropane), 140 (loss of anhydro-acid), 122 (loss of neutral acid), 91 (loss of methylamine)	[39,40,68,69,70]
**Trisubstituted tropane (non-epoxy)**	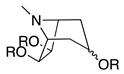	154, 138, 113 (if 3-hydroxyl) 94 (often base peak)	156, 138, 120, sometimes 94, 93 or 91	[37,39,40,64,68,69]
**Epoxytropane**	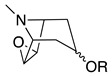	154, 94 (often base peak), 55, 42	156, 138, generally little fragmentation beyond that.	[37,69]
**3-Monosubstituted Nortropane**	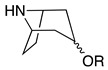	156, 138, 110 (base peak), 80, 68	128 (loss of anhydro-acid), 110 (loss of neutral acid), 93, 91	[40,61,68]
**3-substituted 6,7-Epoxynortropane**	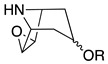	122 (base peak), 124, 94, 80	142, 124 (norscopine)	[61,68,71,72]
**3-substituted 6,7-Dehydrotropane**	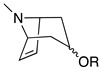	94 (base peak)	140, (loss of anhydro acid) 122 (loss of neutral acid), 93, 91	[64]
**Tigloyl esters**	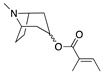	Loss of 99	Loss of 100	[37]
**Tropic acid derivative [(S)-1 or substituted on tropic acid]**	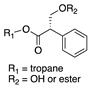	271 (dehydration or elimination of substituent), loss of 30 (formaldehyde via McLafferty rearrangement), 133, 121, 103 (derived from apotropic acid)	272 (dehydration or elimination of substituent), 103, loss of 166.064 or 148.044)	[37,68,69,73]
**Phenylacetate esters**	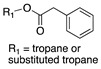	91 (derived from benzyl group)	Loss of 136.05 (neutral acid)	[37,61]
**Pyrrolidine**	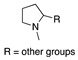	Often low-intensity M^+^; 85, 84 (usually base peak), 83, 82, 70, 55, 32	84 (*N*-methylpyrrolinium ion), loss of 83.	[68]

## Data Availability

Not applicable.

## Supplementary Materials

The following are available online: Table S1: Monosubstituted tropane alkaloids described in *Datura*, Table S2: Disubstituted tropane alkaloids from *Datura*, Table S3: 3,6,7-Trisubstituted Tropane alkaloids from *Datura*, Table S4: 3-Substituted 6,7-epoxytropane alkaloids from *Datura*, Table S5: Nortropanes detected in *Datura*.

## Abbreviations

Aromatic amino acid aminotransferase (ArAT), atmospheric pressure chemical ionization (APCI), coenzyme A (CoA), correlation spectroscopy (COSY), Dalton (Da), desorption ESI (DESI), dimethylaminopurine (cytokinin) (DMAA), direct analysis in real-time (DART), distortionless enhancement via polarization transfer (DEPT), electron ionization (EI), electrospray ionization (ESI), planar electrochromatography or thin-layer electrochromatography (ETLC), gas chromatography-mass spectrometry (GC-MS), heteronuclear multiple bond correlation (HMBC), heteronuclear single quantum coherence (HSQC), high-performance liquid chromatography (HPLC), high-performance TLC (HPTLC), hyoscyamine 6β-hydroxylase (H6H), indole-3-acetic acid (auxin) (IAA), infrared (IR), ion-mobility spectrometry (IMS), laser ablation dart imaging (LADI), liquid chromatography-mass spectrometry (LC-MS), mass spectrometry (MS), methylputrescine oxidase (MPO), multiple reaction monitoring (MRM), tandem mass spectrometry (MS/MS), nuclear magnetic resonance (NMR), nuclear Overhauser effect spectroscopy (NOESY), putrescine *N*-methyltransferase (PMT), quadrupole time-of-flight mass spectrometry (QToF), rotating frame Overhauser enhancement spectroscopy (ROESY), selective reaction monitoring (SRM), time-of-flight mass spectrometry (TOF), thin-layer chromatography (TLC), total correlation spectroscopy (TOCSY), tropinone reductase (TR), ultrahigh-performance liquid chromatography (UHPLC), ultraviolet (UV), vibrational circular dichroism (VCD).
