# Supplementary material for: Alkaloids of the Genus Datura: Review of a Rich Resource for Natural Product Discovery
Source: Molecules. 2021 Apr 30;26(9):2629. doi: 10.3390/molecules26092629 (PMC8124590; doi:10.3390/molecules26092629)
Supplement: Supplementary file 1 [file molecules-26-02629-s001.zip › molecules-1180289-supplementary.pdf]

Review

Alkaloids of the genus *Datura*: review of a rich resource for natural product discovery

Maris A. Cinelli [1*] and A. Daniel Jones [1,*]

Electronic Supplementary Material

Contents:

Table S1: **Monosubstituted Tropane Alkaloids Described in *Datura***
Table S2: **Disubstituted Tropane Alkaloids from *Datura***
Table S3: **3,6,7-Trisubstituted Tropane Alkaloids from *Datura***
Table S4: **3-Substituted 6,7-Epoxytropane Alkaloids from *Datura***
Table S5: **Nortropanes Detected in *Datura***

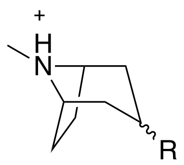

Table S1: Monosubstituted Tropane Alkaloids Described in *Datura*.

R =	Stereochemistry at position 3 (and common name and number)	Species found in	References (see main text for reference numbers)
**OH**	α (tropine, α-**10**)[b]	All (some variation in varieties), including some transformed root cultures	22, 39, 40, 61, 64, 67, 68, 72, 73, 75, 76, 114-115, 118, 128, 130, 146, 152
**OH**	β (Pseudotropine, β-**10**)	*D. inoxia* (including root cultures), *D. discolor*, *D. ferox*, *D. wrightii*, *D. leichhardtii*, *D. stramonium* (including hairy root cultures), *D. ceratocaula*, *D. quercifolia* (root cultures)	22, 39, 40, 61, 64, 67, 68, 73, 113-114, 118, 130, 152
**OAc**	α (α-**24**)	*D. inoxia* (including root cultures), *D. leichardtii* ssp. *pruinosa* (isomer unspecified), *D. stramonium* (isomer unspecified, including hairy root cultures), *D. quercifolia* (root cultures), *D. ceratocaula* (isomer unspecified). *D. wrightii* root cultures	39, 40, 61, 64, 67, 68, 72, 73, 113-114, 118, 128, 146, 152
**OAc**	β (β-**24**)	*D. inoxia*, *D. stramonium* (root cultures), *D. wrightii* root cultures	39, 102, 128
**=O**	N/A (tropinone, **19**)	*D. leichhardtii* and ssp *pruinosa*, *D. metel* (var. *fastuosa), D. inoxia* (including root cultures, *D. stramonium* (including root cultures), *D. querficolia* (root cultures)	22, 40, 61, 67, 68, 72, 75, 83, 89, 114, 118, 146

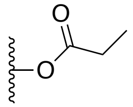	α (α-**25**)	*D. inoxia*	39
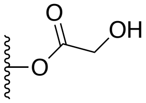	Unspecified, **26**	*D. inoxia*, *D. stramonium*, *D. ferox*, *D. leichhardtii* (both ssp), *D. wrightii*, *D. metel* (including var. *fastuosa*), *D. ceratocaula*,	40, 63, 64, 113, 118, 146
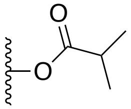	α (α-**27**)	*D. inoxia*	39
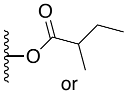 or 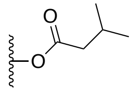	α (α-**28**)[a]	*D. stramonium* (isomer unspecified), *D. inoxia*	39, 64
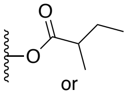 or 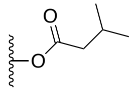	β (β-**29**)[a]	*D. inoxia*	39
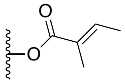	α (α-**23**)	*D. stramonium* (cultures, and plant, isomer unspecified), *D. inoxia* (plant and root cultures), *D. ceratocaula*, *D. stramonium* (var. *tatula*, var. *godronii*), *D. wrightii*, *D. leichhardti*i (both ssp), *D. metel* (including var. *fastuosa*), *D. ferox*	22, 37, 39, 40, 61, 64, 68, 72, 73, 75, 83, 91, 113-115, 118, 128, 151-152
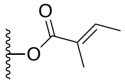	β (tigloidine), β-**23**	*D. inoxia* (including root cultures), *D. ceratocaula*, *D. ferox*, *D. leichhardtii* ssp. *pruinosa*, *D. metel* (including *D. fastuosa*), D. *stramonium*	22, 39, 40, 61, 64, 68, 72, 73, 83, 114

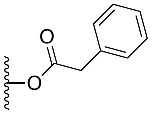	α (or unspecified, α-**30**)	*D. inoxia*, (isomer unspecified, including hairy root cultures) *D. stramonium* (multiple varieties and hairy root cultures), *D. ferox*, *D. wrightii*, *D. leichhardtii* (both ssp), *D. metel* (including var. *fastuosa*), *D. ceratocaula*, *D. quercifolia* (root cultures),	22, 40, 61, 64, 67, 73, 68, 72, 83, 75, 91, 113-115, 140
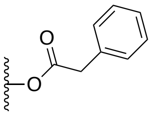	β (β-**30**)	*D. stramonium*	68, 140
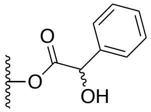	Stereochemistry unspecified, described as just "homatropine" (**31**)	*D. metel*	41
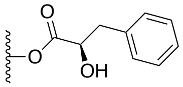	α (littorine), **11**	All except *D. quercifolia*, including hairy root cultures	32, 40, 64, 68, 72, 73, 89, 109, 118, 130, 146, 152
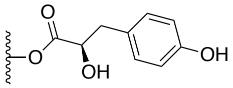	α (presumably), **32**	*D. inoxia*, *D. stramonium*	64, 113
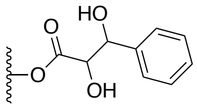	Unspecified, **33**	*D. inoxia*	146
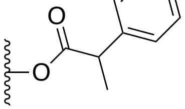	Unspecified, **34**	*D. inoxia*, *D. stramonium*	64, 68, 72
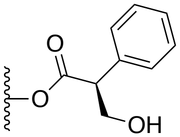	α (hyoscyamine, or reported as "atropine"), (*S*)-**1** or **1**	All species, including transformed root cultures	22, 40, 36, 39, 61, 64, 68, 72, 73-75, 91, 115, 128, 130, 140, 146, 151-152
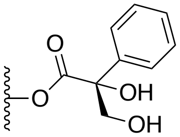	Not specified, but presumably α, **35**	*D. stramonium*, *D. wrightii*, *D. ceratocaula*, *D. inoxia*	40, 68, 73, 91, 146, 151.

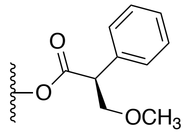	Not specified, but presumably α, **36**	*D. inoxia* (including hairy root cultures), *D. stramonium*	61, 64, 68, 75, 113
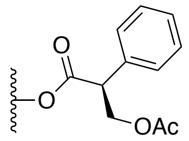	Not specified, but presumably α, **37**	*D. stramonium* (multiple varieties), *D. ferox*, *D. inoxia*, *D. wrightii*, *D. leichhardtii* (both ssp), *D. metel* (including var. fastuosa), *D. ceratocaula*	22, 32, 40, 64, 68, 72, 73, 114, 151
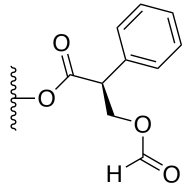	α (α-**38**)	*D. quercifolia*	73
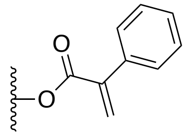	Not specified, but presumably α (α-**39**)	All except *D. discolor*, including multiple transformed root cultures	22, 39, 40, 41, 61, 64, 67, 68, 72, 73, 75, 83, 91, 114-115, 118, 151-152
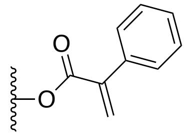	β (proposed), β-**39**	*D. stramonium*	151
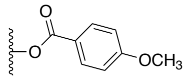	α (datumetine), **8**	*D. metel*	45

[a]Some references (e.g., 39) refer to separate isomers as "2-methylbutyryloxy"- and "methylbutyryloxy". These are presumably valeroyl and isovaleroyl and are classified as such. [b.]7-hydroxytropane is reported in reference 140 – there do not appear to be any other references to this compound.

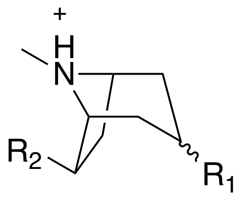

Table S2: Disubstituted Tropane Alkaloids from *Datura*.

R_1 =	R_2 =	Stereochemistry at Position 3 and number	Species found in	References (see main text for reference numbers)
**OH**	**OH**	Not specified, **41**	*D. inoxia*, *D. metel* var. *fastuosa*, *D. ceratocaula*, *D. stramonium*	22, 40, 64, 83, 113
**OAc**	**OH**	Not specified, **44**[b]	*D. inoxia* (including root cultures), *D. stramonium* (multiple varieties, including root cultures, *D. ceratocaula*, *D. wrightii* (root cultures)	61, 64, 68, 72, 73, 91, 113-115, 118, 128, 151
**OH**	**OAc**	Not specified, specified as α in *D. metel*, **45**	*D. stramonium*, *D. metel* var. *fastuosa*, *D. quercifolia* (root cultures)	40, 64, 68, 72, 128
**OAc**	**OAc**	Not specified, **42**	*D. ceratocaula*, *D. stramonium* (including root cultures), *D. metel* (including var. *fastuosa*), *D. quercifolia*, *D. ferox*, *D. inoxia*,	40, 64, 68, 72, 73, 113, 118, 128,

**OH**	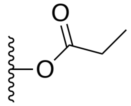	Not specified, **46**	*D. metel*, including var. *fastuosa*	40
**OH**	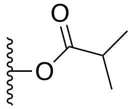	Not specified, **47**	*D. stramonium*, *D. inoxia*	64, 113
**OAc**	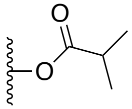	Not specified, **48**	*D. stramonium*	68, 72
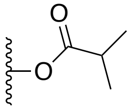	**OH**	Not specified, **49** [a]	*D. inoxia*, *D. wrightii*	40, 114
**OH**	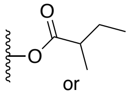 or 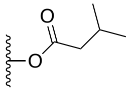	Not specified, **50** [a]	*D. inoxia* (including hairy root cultures), *D. metel* var. *fastuosa*, *D. wrightii*, *D. ceratocaula*, *D. stramonium*	40, 49, 61, 64, 68, 72, 73
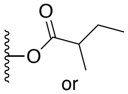 or 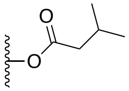	**OH**	Not specified, **51**	*D. inoxia*, *D. metel* var. *fastuosa*, *D. ferox*, *D. stramonium* (multiple varieties)	40, 64, 68, 113-114
**OH**	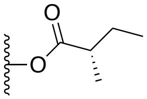	α, α-**9**	*D. ceratocaula*	49

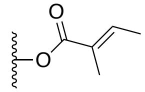	**OH**	Not specified, or α, α-**52**	*D. stramonium* (multiple varieties), *D. ferox*, *D. inoxia* (including hairy root cultures), *D. wrightii*. *D. ceratocaula*, *D. stramonium hairy root cultures*, *D. leichhardtii* (both ssp.), *D. metel* (including var. *fastuosa*)	22, 40, 49, 61, 64, 68, 72, 73, 75, 91, 113-114, 118, 128, 140, 146, 151
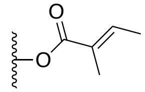	**OH**	β, β-**52**	*D. ceratocaula*, *D. stramonium*	68, 72, 73
**OH**	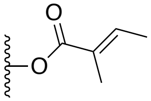	Not specified or α (α-**53**)	*D. inoxia* (including hairy root cultures), *D. stramonium* (multiple varieties), *D. ceratocaula*, *D. ferox*, *D. wrightii*, *D. leichhardtii* (both ssp.), *D. metel* (including var. *fastuosa*)	22, 49, 61, 64, 68, 72, 73, 75, 91, 113-115, 128, 140, 151
**OH**	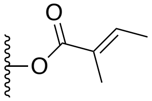	β (β-**53**)	*D. stramonium* (multiple varieties), *D. inoxia*	22, 64, 113
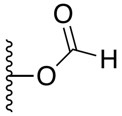	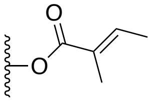	Not specified or α (**54**)	*D. stramonium*	68
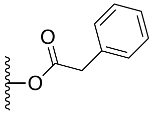	**OH**	Not specified, but presumably α (**55**)	*D. stramonium*, *D. inoxia*	40, 64, 68, 72, 113
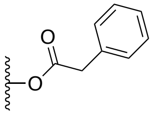	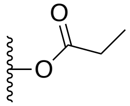	α (α-**56**)	*D. stramonium*	68

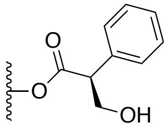	**OH**	Not specified, but presumably α, **40**	*D. stramonium* (multiple varieties), *D. inoxia* (including transformed root cultures), *D. wrightii*, *D. leichhardtii* (both ssp.), *D. metel* (including var. *fastuosa*), *D. ceratocaula*, *D. ferox*	36, 40, 61, 64, 68, 72, 73, 75, 91, 113-115, 128, 146, 151
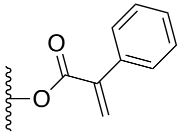	**OH**	Not specified, or α, **57**	*D. stramonium*, *D. ceratocaula*, *D. inoxia* (including hairy roots), *D. wrightii*, *D. leichhardtii* (both ssp), *D. metel*	40, 61, 64, 68, 72, 73, 75, 83, 113
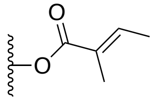	**OAc**	α, α-**58**	*D. inoxia*, *D. metel* var. *fastuosa*, *D. leichhardtii* (both ssp.), *D. ceratocaula*, *D. stramonium* (including root cultures), *D. discolor* (possibly)	40, 49, 64, 68, 72, 114, 118,
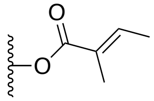	**OAc**	β, β-**58**	*D. ceratocaula*	40
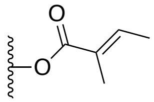	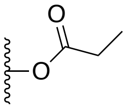	Not specified, or α, **59**	*D. inoxia*, *D. metel* (including var. *fastuosa*), *D. stramonium* (multiple varieties), *D. ferox*, *D. wrightii*, *D. leichhardtii* (both ssp.)	22, 40, 49, 64, 68, 72, 114

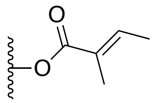	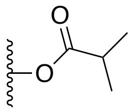	Not specified, or α, **60**	*D. stramonium* (multiple varieties and hairy root cultures), *D. inoxia*, *D. ferox*, *D. wrightii*, *D. leichhardtii* ssp. *pruinosa*, *D. metel* (including var. fastuosa)	22, 64, 68, 72, 113-115, 118, 151
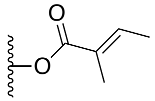	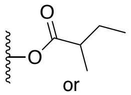 or 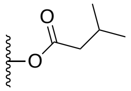	α, α-**61**[a]	*D. stramonium* (multiple varieties and hairy root cultures), *D. ferox*, *D. inoxia*, *D. wrightii*, *D. leichhardtii* (both ssp.), *D. metel* (including var. fastuosa)	22, 32, 40, 49, 64, 68, 72, 114, 140, 151
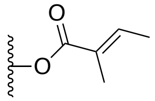	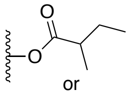 or 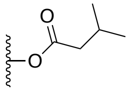	β, β-**61**[a]	*D. stramonium*	68
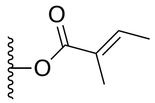	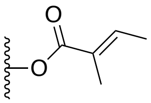	Not specified, or α, α-**43**	*D. stramonium* (multiple varieties and hairy root cultures), *D. ferox*, *D. inoxia* (including hairy root cultures), *D. wrightii*, *D. leichhardtii* (both ssp.), *D. metel* (including var. *fastuosa*), *D. ceratocaula*, *D. discolor*	22, 40, 49, 61, 64, 68, 72, 113, 128, 140, 146, 151-152
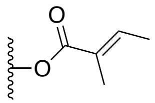	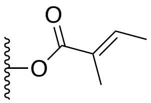	β, β-**43**	*D. inoxia* (including hairy root cultures) *D. wrightii*, *D. stramonium* (multiple varieties)	40, 61, 68, 72, 114
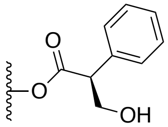	**OAc**	Not specified, but presumably α, **62**	*D. stramonium*	64, 68, 72, 83

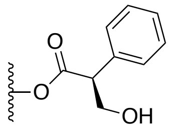	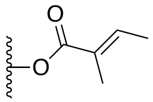	Not specified, but presumably α, **63**	*D. inoxia* (including hairy roots), *D. ceratocaula*, *D. stramonium*	40, 49, 61, 64, 68, 73, 91, 113-114, 151
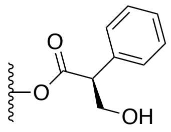	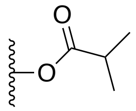	Not specified, but presumably α, **64**	*D. stramonium*	64
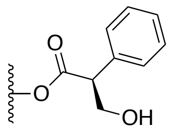	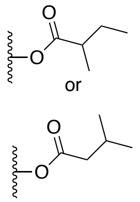	Not specified, but presumably α, α-**65**	*D. inoxia* (including hairy root cultures), *D. metel* var. *fastuosa*, *D. stramonium*	40, 49, 61, 64, 114
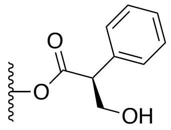	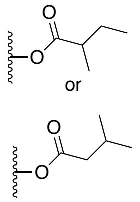	β, β-**65**	*D. stramonium*	64

[a]Some references (e.g., 49) refer to separate isomers as "2-methylbutyryloxy"- and "methylbutyryloxy". These are presumably valeroyl and isovaleroyl and are classified as such.
[b]Sometimes referred to as "6-hydroxyacetoxytropane (e.g., reference 72).

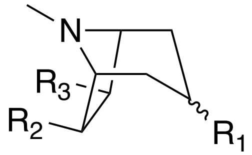

Table S3: 3,6,7-Trisubstituted Tropane Alkaloids from *Datura.*

R_1 =	R_2 =	R_3 =	Stereochemistry at Position 3	Species found in	References (see main text for reference numbers)
**OH**	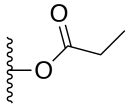	**OH**	Not specified, **67**	*D. stramonium*	64
**OAc**	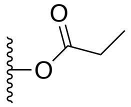	**OH**	Not specified, **68**	*D. inoxia*	113
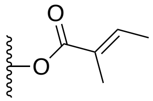	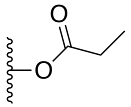	**OH**	Not specified, presumably α, α-**69**	*D. stramonium* (including hairy root cultures), *D. ferox*, *D. inoxia*, *D. wrightii*, *D. leichhardtii* (both ssp), *D. metel* (including var. *fastuosa*), *D. ceratocaula*	22, 40, 64, 68, 72, 73, 113, 118

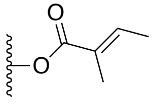	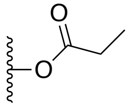	**OH**	Not specified, possibly β, β-**69**	*D. inoxia*, *D. stramonium*	68, 114
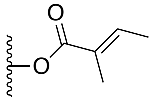	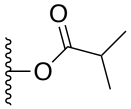	**OH**	Not specified, **70**	*D. stramonium*	64
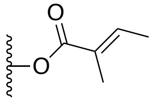	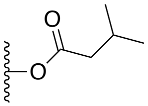	**OH**	α or unspecified, α-**71**	*D. stramonium* (multiple varieties), *D. ferox*, *D. inoxia*, *D. wrightii*, *D. leichhardtii* (both ssp), *D. metel* var. *fastuosa*, *D. ceratocaula*	22, 37, 40, 64, 68, 91, 113, 140
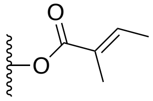	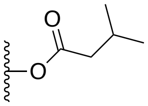	**OH**	β, β-**71**	*D. stramonium* (multiple varieties and hairy root cultures)	22, 64, 68, 72, 91, 140, 151
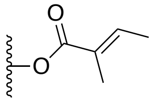	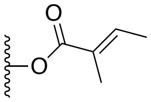	**OH**	α or unspecified (α-**72**)	*D. stramonium* (multiple varieties and hairy root cultures), *D. ceratocaula*, *D. ferox*, *D. inoxia* (including hairy root cultures), *D. wrightii*, *D. leichhardtii* (both ssp), *D. metel* (including *D. fastuosa*), *D. discolor*, *D. quercifolia* (hairy roots)	22, 32, 39, 40, 61, 64, 68, 72, 73, 91, 114, 118, 128, 130, 140, 146, 152

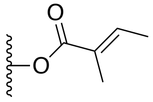	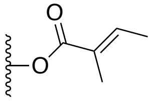	**OH**	β, β-**72**	*D. inoxia*, *D. stramonium*	40, 68, 72, 91, 151
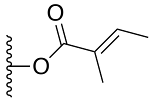	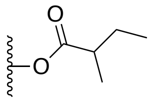	**OH**	α-**73**	*D. stramonium*	39
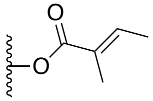	**OH**	**OH**	(meteloidine), α-**20**	*D. wrightii*, *D. leichhardtii* (ssp. *pruinosa*), *D. metel* (including *D. fastuosa*, *D. ceratocaula*, *D. inoxia*, *D. stramonium* (including root cultures), *D. ferox*	32, 39, 40, 64, 72, 73, 91, 114, 140, 146, 151-152
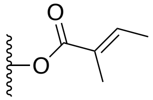	**OH**	**OH**	β, β-**20**	*D. ceratocaula*	73
**OH**	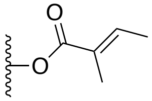	**OH**	Unspecified, **74**	*D. inoxia*, *D. stramonium*	40, 72, 146
**OH**	**OH**	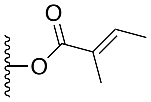	Unspecified, **75**	*D. inoxia*	114

**OH**	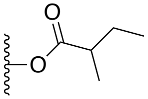	**OH**	Unspecified, **76**	*D. stramonium*	64
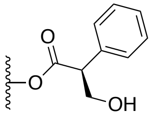	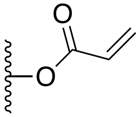	**OH**	α, α-**77**	*D. ferox*	37
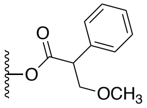	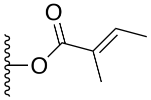	**OH**	β, β-**7**	*D. stramonium*	48

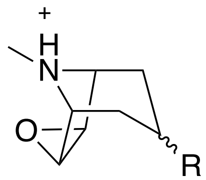

Table S4: 3-Substituted 6,7-Epoxytropane Alkaloids from *Datura.*

R_1 =	Stereochemistry at Position 3 and number	Species found in	References (see main text for reference numbers)
**OH**	α (scopine, **78**)	*D. inoxia*, *D. stramonium*	40, 64, 68, 75, 113-114
**OAc**	Unspecified, **79**	*D. inoxia*	113
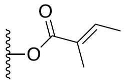	Unspecified, **80**	*D. inoxia*	113

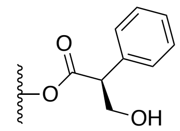	α (Scopolamine, **2**)	All species, including hairy root cultures	22, 32, 37, 39, 40, 41, 61, 64, 67, 68, 72, 75, 81, 83, 89, 113-115, 118, 128, 130, 140, 146, 151
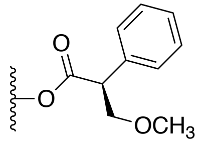	α (methylscopolamine, **81**)	*D. metel* var. *fastuosa*, *D. stramonium*, *D. inoxia* (including hairy root cultures)	40, 64, 68, 113, 151,
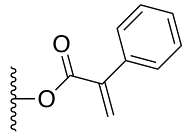	Unspecified or α (aposcopolamine, **82**)	*D. ceratocaula*, *D. stramonium* (multiple varieties), *D. ferox*, *D. inoxia*, *D. wrightii*, *D. leichhardtii* ssp. *leichhardtii*, *D. metel* (including var. *fastuosa*)	22, 31, 37, 40, 41, 64, 68, 72, 73, 75, 83, 113-114, 118 151
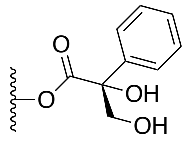	Unspecified or α, (anisodine, **22**)	*D. wrightii*	40
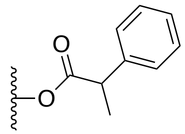	Unspecified or α, **83**	*D. inoxia*, *D. stramonium*	64, 113
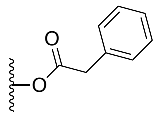	Unspecified or α (**84**)	*D. stramonium* (including root cultures), *D. inoxia*, *D. ferox*	37, 40, 64, 68, 72, 75, 113, 118

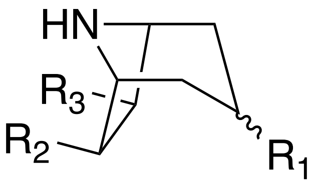

Table S5: Nortropanes Detected in *Datura*.

R_1 =	R_2 =	R_3 =	Stereochemistry at Position 3	Species found in	References (see main text for reference numbers)
**OAc**	**H**	**H**	Not specified, **91**	*D. wrightii* (root cultures), *D. quercifolia* (root cultures)	83, 128
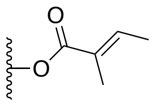	**H**	**H**	Not specified, **92**	*D. stramonium*, *D. inoxia* (including hairy roots)	40, 61, 68

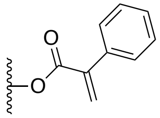	**H**	**H**	Not specified, presumably α, **93**	*D. stramonium*, *D. inoxia*, *D. ceratocaula*	40, 68, 73, 113-114
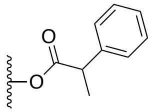	**H**	**H**	Not specified, **94**	*D. stramonium*, *D. inoxia*	68, 113
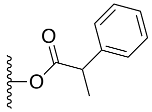	**6,7-epoxy**	**6,7-epoxy**	Not specified, **95**	*D. inoxia*	113
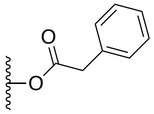	**6,7-epoxy**	**6,7-epoxy**	Not specified, **96**	*D. stramonium*, *D. ferox*, *D. inoxia*, *D. wrightii*, *D. leichhardtii* spp. *leichhardtii*, *D. metel*, *D. ceratocaula*	40, 68, 72
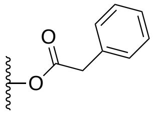	**H**	**H**	Not specified, **97**	*D. inoxia*, *D. stramonium*	113
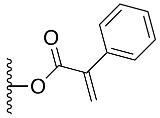	**6,7-epoxy**	**6,7-epoxy**	Not specified, **98**	*D. stramonium*, *D. inoxia*, *D. ferox*, *D. wrightii*, *D. leichhardtii* spp. *leichhardtii*, *D. metel* (including var. *fastuosa*), *D. ceratocaula*	40, 64, 68, 72, 113

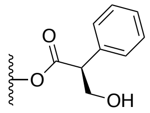	**6,7-epoxy**	**6,7-epoxy**	α or unspecified, **99**	*D. stramonium*, *D. discolor*, *D. inoxia*, *D. ferox*, *D. metel* var. *fastuosa*, *D. ceratocaula*	40, 68, 130, 151-152
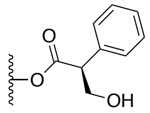	**H**	**H**	α or unspecified, **100**	*D. stramonium*, *D. inoxia*, *D. metel* var. *fastuosa*, *D. ceratocaula*	68, 73, 113, 146, 152
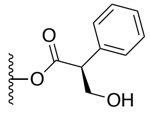	**OH**	**H**	α or unspecified, **101**	*D. inoxia*	146
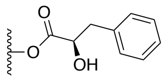	**H**	**H**	Unspecified, **102**	*D. inoxia*	146
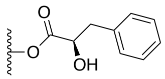	**OH**	**H**	Unspecified, **103**	*D. inoxia*	146

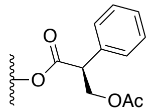	**6,7-epoxy**	**6,7-epoxy**	Unspecified, **104**	*D. stramonium*	68
